# Synthesis of a binary alloy nanoparticle catalyst with an immiscible combination of Rh and Cu assisted by hydrogen spillover on a TiO_2_ support†

**DOI:** 10.1039/c9sc05612b

**Published:** 2020-04-01

**Authors:** Shinya Masuda, Kazuki Shun, Kohsuke Mori, Yasutaka Kuwahara, Hiromi Yamashita

**Affiliations:** Division of Materials and Manufacturing Science, Graduate School of Engineering, Osaka University 2-1 Yamadaoka, Suita Osaka 565-0871 Japan mori@mat.eng.osaka-u.ac.jp yamashita@mat.eng.osaka-u.ac.jp +81-6-6879-7457 +81-6-6879-7457; Unit of Elements Strategy Initiative for Catalysts Batteries (ESICB), Kyoto University Katsura Kyoto 615-8520 Japan

## Abstract

This work demonstrated the use of TiO_2_ as a promising platform for the synthesis of non-equilibrium RhCu binary alloy nanoparticles (NPs). These metals are regarded as immiscible based on their phase diagram but form NPs with the aid of the significant hydrogen spillover on TiO_2_ with concurrent proton–electron transfer. The resulting RhCu/TiO_2_ exhibited 2.6 times higher catalytic activity than Rh/TiO_2_ during hydrogen production from the hydrolysis of ammonia borane (AB), due to a synergistic effect. Theoretical simulations showed a higher energy value for the adsorption of AB on the RhCu alloy and a lower activation energy for the rate determining N–B bond dissociation by the attack of H_2_O during AB hydrolysis compared to monometallic Rh. High-angle annular dark-field scanning transmission electron microscopy and energy-dispersive X-ray spectroscopy confirmed the formation of RhCu alloy NPs with a mean diameter of 2.0 nm on the TiO_2_. H_2_-temperature programmed reduction and *in situ* X-ray absorption fine structure analyses at elevated temperature under H_2_ demonstrated that Rh^3+^ and Cu^2+^ precursors were simultaneously reduced only on the TiO_2_ support. This effect resulted from the improved and limited reducibility of Cu^2+^ and Rh^3+^, respectively. The rate of hydrogen spillover of TiO_2_ is faster as compared to γ-Al_2_O_3_ and MgO as evidenced by sequential H_2_/D_2_ exchanges during *in situ* Fourier transform infrared analyses. Density functional theory calculations also showed that the migration of H atoms on TiO_2_ proceeds with a lower energy barrier than that on Al_2_O_3_, and the reduction of Cu^2+^ species is facilitated by H spillover on the support rather than by direct reduction by H_2_. These results confirm the vital role of TiO_2_ in the formation of the alloy and may represent a new strategy for the synthesis of different non-equilibrium solid solution alloys.

## Abstract 2

This work demonstrates the use of TiO_2_ as a promising platform for the synthesis of non-equilibrium binary solid solution alloy nanoparticles with a combination of immiscible Rh and Cu assisted by the hydrogen spillover.

## Graphical abstract

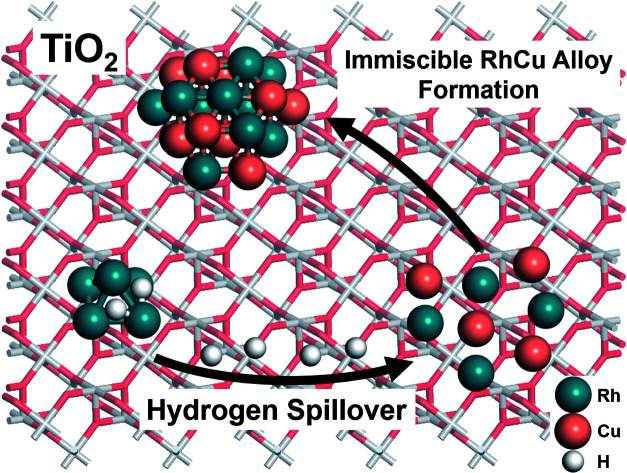

## Introduction

Over the past decade, metal nanoparticles (NPs) have been studied intensively with regard to a wide range of potential industrial,^1,2^ biomedical,^3–5^ electronic,^6,7^ and catalytic applications.^8–11^ This is due to the unique properties of NPs, which are different from those of bulk metals, and the small size effects they exhibit.^12^ In addition, binary alloys, whose nanostructure can consist of solid solution, segregated, or core–shell structures, have been widely researched as advanced catalysts because they show superior catalytic activity and selectivity compared to single metal NPs.^12–17^ Various geometric and electronic effects can appear as a result of differences in certain characteristics of each metal, such as work functions, ionization potentials, and electronegativity.^14,18,19^ These factors can, in turn, have significant effects on the properties of binary alloys. Therefore, the formulations of binary alloys are a vital factor in terms of optimizing their performance.

The replacement of expensive noble metals with low-cost base metals is also an important consideration that affects the economic aspects of these materials. However, there are several combinations of noble and base metals, such as Au–Co, Ru–Ni,^20^ and Rh–Cu,^21^ that are difficult to process into solid solution alloys based on their phase diagram. Thus, even though such combinations have unexplored potential, the catalytic properties of these solid solution alloys have not yet been elucidated. In this regard, Kitagawa *et al.* have reported the use of a conventional procedure to synthesize solid solution alloys with immiscible combinations of metals using a polyol method.^17,21,22^ In addition, Humphrey *et al.* also successfully synthesized solid solution alloys by employing a combination of the polyol method and microwave heating.^23,24^ These research groups demonstrated that the resulting binary alloys with immiscible combinations show interesting performance and characteristics for specific catalytic reactions. The development of new and facile techniques for the synthesis of such solid solution alloys with immiscible metal combinations remains desirable, even though these NPs are not thermodynamically stable and readily grow to form larger particles.^25,26^ One means of mitigating this problem is to fix the NPs on supports having relatively high surface areas, such as metal oxides, so as to maintain their small sizes particularly in the field of catalysis. Interestingly, metal NPs are affected by the supports employed during synthesis and will exhibit different properties depending on the support.^26–28^ Therefore, the selection of an appropriate support for use during the growth of metal NPs is very important, and it is crucial to understand the phenomena that occur on various supports.^29^

Hydrogen spillover is one such phenomenon that is of interest in heterogeneous catalysis. This process involves the surface migration of dissociated H atoms from noble metal particles to nonmetal supports.^30–36^ This spillover effect has been widely studied^30,34–36^ and applied to catalytic systems and the fabrication of hydrogen storage materials.^37–43^ Several types of hydrogenation have been identified in which substrates adsorbed on supports are hydrogenated by activated H atoms generated on metal catalysts and spilled over to supports.^38,42^ Hydrogen spillover has also emerged as a promising strategy for high-density hydrogen storage under near-ambient conditions, based on the utilization of carbon-based materials,^39,43^ metal–organic frameworks,^40,44^ and zeolites.^45^ Hydrogen spillover is initiated by the formation of protons (H^+^) and electrons (e^−^) from H atoms at metal–support interfaces.^30^ The protons diffuse to O_2_^−^ anions to form O–H and H–O–H bonds on the support surface, whereas the electrons simultaneously promote the partial reduction of metals in reducible metal oxides.^46,47^ In the case of a TiO_2_ support, Ti^4+^ ions are partially reduced to Ti^3+^ by the electrons produced by activated H atoms that migrate through the TiO_2_ lattice by exchange between Ti^3+^–Ti^4+^ pairs.^48^ Consequently, hydrogen spillover rarely occurs on non-reducible supports, since the concurrent transfer of protons and electrons will not proceed on such materials.

Our group has previously reported that a combination of Ru and Ni (which are essentially immiscible at equilibrium due to the positive enthalpy of formation for their solid solution alloy) will form a solid solution alloy on a TiO_2_ support when using conventional impregnation and hydrogen reduction procedures.^20,49^ This highly specific formation of the binary alloy is believed to involve intraparticle hydrogen spillover from the Ru surface to the Ni surface on the TiO_2_ support. This synthesis can be performed without using a polymeric surfactant, and the strong metal–support interaction obtained from the TiO_2_ support allows the loading of small size NPs of the alloy.^50,51^ However, the mechanism by which solid solution alloys are generated from immiscible metals on a TiO_2_ support and the applicable metal combinations remain unclear.

In the work reported herein, we determined that a combination of Rh and Cu, which are also thermodynamically immiscible according to the associated phase diagram (Fig. S1†),^52^ forms binary solid solution alloy NPs on a TiO_2_ support. These NPs exhibit superior catalytic performance compared to monometallic Rh during the hydrolysis of ammonia borane (AB) owing to a significant synergistic effect obtained from the two metals. The present study elucidated the specific mechanism responsible for the alloy formation based on *in situ* characterization by means of H_2_ temperature-programmed reduction (TPR), X-ray adsorption fine structure (XAFS) analysis, and Fourier transform infrared (FTIR) spectroscopy. Density functional theory (DFT) calculations were also performed to clarify not only the promotional effect of the RhCu alloy but also the enhanced hydrogen spillover occurring on the TiO_2_ support.

## Experimental

### Materials

Rutile TiO_2_ (JRC-TIO-6) and anatase TiO_2_ (JRC-TIO-8) were kindly supplied by the Catalysis Society of Japan. MgO was purchased from Wako Pure Chemical Industries, Ltd. Ethanol, RhCl_3_, CuCl_2_·2H_2_O, and ZrO_2_ were purchased from Nacalai Tesque. γ-Al_2_O_3_ was obtained from Strem Chemicals, Inc. Ammonia borane (AB, NH_3_BH_3_) was obtained from the Aldrich Chemical Co. All commercially available compounds were used as received. Distilled water was employed as the reaction solvent.

### Preparation of catalysts

TiO_2_ (0.5 g) was added to a mixture of distilled water (50 mL) and ethanol (50 mL), followed by the addition of RhCl_3_ (21.0 mg) and CuCl_2_·2H_2_O (17.1 mg). This mixture was stirred at room temperature for 1 h, after which the solvents were evaporated under vacuum. Finally, the sample was reduced with H_2_ at a heating rate of 5 °C min^−1^ (20 mL min^−1^, 350 °C) for 2 h to yield RhCu/TiO_2_ (Rh 2.0 wt%; Rh : Cu = 1 : 1). RhCu/ZrO_2_, RhCu/γ-Al_2_O_3_, and RhCu/MgO with the same Rh and Cu loadings were also synthesized according to the same procedure.

### Characterization

Transmission electron microscopy (TEM) micrographs were obtained with a field emission (FE) TEM instrument (Hf-2000, Hitachi). Scanning transmission electron microscopy (STEM), elemental mapping and line analysis were performed using a JEOL-ARM 200F instrument equipped with a Kevex EDX detector (JED 2300T) operated at 200 kV. Temperature-programmed reduction with H_2_ (H_2_-TPR) was conducted using a BEL-CAT (BEL Japan, Inc.) instrument by heating 30 mg samples at 5 °C min^−1^ from 50 to 600 °C under a 5.0% H_2_/Ar flow. In these experiments, as-deposited samples before H_2_ reduction were employed. Rh K-edge and Cu K-edge *in situ* X-ray absorption fine structure (XAFS) spectra were recorded in the transmission mode at the 01B1 beamline station in conjunction with a Si (111) monochromator at SPring-8, JASRI, Harima, Japan (proposal numbers 2017A1057 and 2017B1084). In a typical experiment, spectra were acquired while a pellet sample was held in a batch-type *in situ* XAFS cell. XAFS data were processed using the ATHENA code (Demeter).^53^ The Fourier transformation (FT) of *k*^3^-weighted normalized extended XAFS (EXAFS) data was performed over the range of 3.0 Å < *k*/Å^−1^ < 12 Å to obtain the radial structure function.

### Hydrolysis of AB

In a typical experiment, a quantity of the RhCu/TiO_2_ catalyst (20 mg) combined with 8 mL water was transferred into a Schlenk-type reaction vessel (30 mL) connected to a gas buret. After the system was purged three times with Ar, 2 mL of a 2 mmol aqueous solution of AB was added to the vessel and allowed to react at 30 °C. The reaction was considered to start at the moment that the AB solution was added to the vessel and the reaction progress was monitored periodically. TOF values were defined as (H_2_ mol)/((total Rh mol) min).

### Evaluation of hydrogen spillover using *in situ* FTIR

*In situ* Fourier transform infrared (FTIR) spectra were acquired using a JASCO FT/IR-6600 instrument over the range of 4400–400 cm^−1^. In each case, an approximately 30 mg quantity of the sample (the as-prepared Rh supported catalyst) was pressed into a self-supported wafer (*ϕ*10 mm). This wafer was subsequently mounted in a specially designed quartz IR cell (Makuhari Rikagaku Garasu Inc.) equipped with a water-cooling condenser and an electric heater and connected to a gas-exchange system. Spectra were acquired using a previously reported procedure.^54^ The specimen was first reduced at 350 °C under a H_2_ flow (20 mL min^−1^) for 2 h. Following this, the sample was heated under N_2_ at 150 °C for 1 h to remove physisorbed water (after which no further changes in the spectrum were observed) and the baseline spectrum was collected.

Spectra were also obtained after the sample was heated under a H_2_ atmosphere at 50 °C for 30 min, to ensure equilibration. Following this initial heating step, the gas flow was switched to *D*_2_ for 10 min and then back to H_2_ for 10 min. This switching process was repeated again and the resulting three-dimensional changes over time in the peak at 1200 cm^−1^ (attributed to the *δ*_D–O–D_ stretching vibration) were monitored.

### DFT calculations

All density functional theory (DFT) calculations were performed with the DMol3 program in the Materials Studio 17.2 software package.^55,56^ The generalized gradient approximation (GGA) exchange-correlation functional proposed by Perdew, Burke, and Ernzerhof (PBE) was used to obtain a better understanding of the semiconductor electronic properties of these materials. This function was combined with the double-numerical basis set plus polarization functions (DNP) in conjunction with a cutoff value of 4.0 Å. We adopted the medium level in the DMol^3^ program for the integration grid. Rh_24_ and Rh_12_Cu_12_ clusters with their (111) facets exposed were selected as models of monometallic and binary clusters, respectively, with the bottom two layers fixed at the corresponding bulk positions and the top layers allowed to relax during geometry optimizations. Adsorption energy values (*E*_ad_) were estimated from the differences between the optimized energies of individual substances and the combined cluster model and AB. *E*_ad_ was defined as *E*_adsorbate_ + *E*_cluster_ − *E*_adsorbate/cluster_, where *E*_adsorbate_ is the total energy of the free adsorbate, *E*_cluster_ is the total energy of the bare cluster and *E*_adsorbate/cluster_ is the total energy of the adsorbate–cluster system. These activation energy calculations were based on a process previously reported by Peng *et al.*^57^

Simulations of the reduction of Cu–O groups *via* H_2_ spillover were performed using a TiO_2_(101) plane with atomic thickness and a unit cell of 10.929 × 9.188 × 16.394 Å together with a hexagonal Al_2_O_3_(100) plane with atomic thickness and a unit cell of 9.518 × 12.991 × 14.277 Å. In addition, a γ-Al_2_O_3_ (100) plane with atomic thickness and a unit cell of 16.826 × 16.136 × 24.318 Å was also used for calculations. Periodic boundary conditions were applied, with some of the top-layer atoms allowed to relax during geometry optimizations and the other layers fixed at the corresponding bulk positions. The slab was separated by a vacuum space with a height of 20 Å and tetrahedral Rh_5_ clusters were loaded on the surface of each oxide when modeling H_2_ dissociation.

## Results and discussion

### Synergistic catalytic effect of RhCu/TiO_2_

Various Rh, Cu, and RhCu supported catalysts were prepared using a conventional impregnation method. Briefly, aqueous solutions of RhCl_3_ and CuCl_2_·2H_2_O containing ethanol were deposited on various supports, including TiO_2_ (rutile: R), TiO_2_ (anatase: A), γ-Al_2_O_3_, ZrO_2_, and MgO. The resulting samples were subsequently reduced under a H_2_ flow (20 mL min^−1^) at 350 °C, without applying a prior calcination step.

**Fig. 1:**
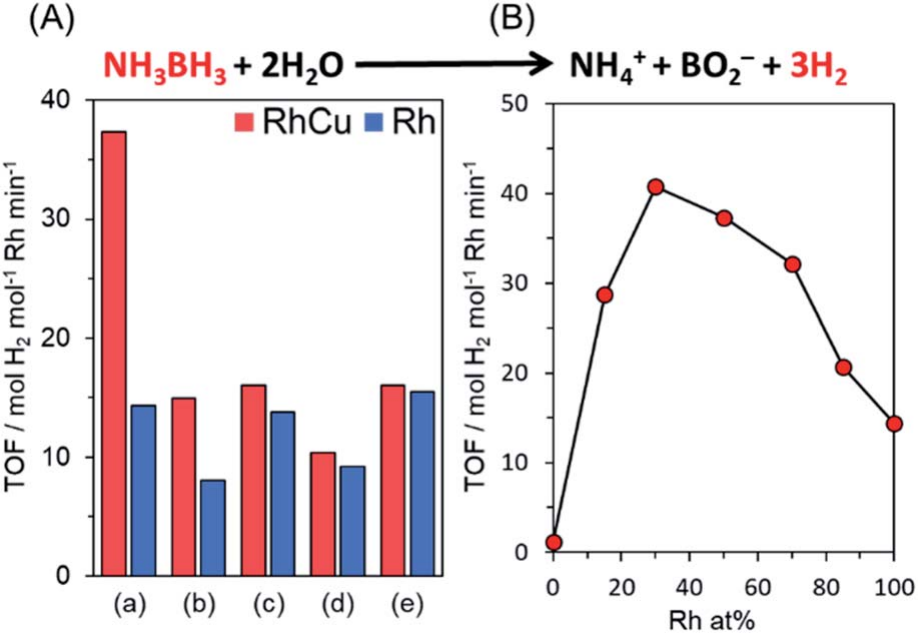
(A) TOF values for hydrogen production from AB hydrolysis over Rh and RhCu supported catalysts on (a) TiO_2_ (rutile), (b) TiO_2_ (anatase), (c) γ-Al_2_O_3_, (d) ZrO_2_ and (e) MgO and (B) TOF values obtained using RhCu supported TiO_2_ (R) catalysts with different Rh : Cu ratios (catalytic conditions: catalyst 20 mg, 10 mL 0.2 M aqueous AB solution, 30 °C).

The catalytic performance of each sample was assessed by monitoring hydrogen production during the hydrolysis of AB. AB was used because this compound has emerged as one of the most practical hydrogen storage compounds due to its high stability as a solid under ambient conditions and superior hydrogen storage capacity (19.6 wt%).^49,58–60^Fig. 1A provides the turnover frequency (TOF) values for various Rh and RhCu supported catalysts, while the time courses of reactivities are summarized in Fig. S2.† The RhCu/TiO_2_ (R) showed the highest catalytic activity, which was 2.6 times that of a single Rh catalyst, while the other RhCu supported catalysts did not show any significant enhancement as compared to the corresponding Rh catalysts, and the activities of the Cu supported catalysts were negligible (Fig. S2†). The RhCu/TiO_2_ (R) exhibited the highest promotional alloying effect (Fig. 1B) when the atomic ratio of Rh and Cu was adjusted to 7 : 3. The volcano-type relationship between activity and composition clearly suggests the formation of a solid solution RhCu alloy structure on the TiO_2_ and an unusual synergistic effect originating from the combination of Rh and Cu. Transmission electron microscopy (TEM) images of Rh and RhCu NPs supported on TiO_2_ (R) (Fig. S3†) also indicate that the improved activity of the RhCu was not due to the size of the NPs, since the average diameters of the Rh and RhCu NPs were approximately 1.9 and 2.0 nm, respectively.

In addition, when Rh/TiO_2_ (R) and RhCu/TiO_2_ (R) specimens were calcined at 500 °C prior to the H_2_ reduction trials, both specimens showed the same level of activity (Fig. S4†). This effect is attributed to the separation of Rh and Cu ions at high temperatures. From these results, it is evident that a unique RhCu binary alloy was formed on the TiO_2_ support during simple H_2_ reduction at 350 °C. These catalysts supported on TiO_2_ (R) were subsequently characterized, as described below (in which TiO_2_ (R) is simply referred to as TiO_2_).

To investigate the alloying effect of Rh and Cu during AB hydrolysis, DFT calculations were performed based on Rh_24_ and Rh_12_Cu_12_ clusters. During these calculations, the level of the highest occupied molecular orbital (HOMO) could be tuned through the coupling of the electronic structure as the cluster composition was varied. When a portion of the Rh_24_ cluster was replaced with Cu metal, the HOMO level was found to be raised from −4.069 eV (Rh_24_) to −3.977 eV (Rh_12_Cu_12_). This result suggests that the RhCu alloy is theoretically more electron-rich than monometallic Rh, and this finding is supported by Rh 3d X-ray photoelectron spectroscopy (XPS) analysis of the Rh/TiO_2_ and RhCu/TiO_2_ (Fig. S5†). In these data, the RhCu/TiO_2_ XPS peak is shifted to a lower binding energy compared to that generated by the Rh/TiO_2_. This shift is attributed to a change in the electronic state of Rh due to electron donation from the Cu. Charge transfer from Cu atoms to Rh atoms occurs because of the respective electronegativity values of Rh and Cu (2.28 and 1.90). Note that there is no clear difference in the Cu 2p XPS spectra of Cu/TiO_2_ and RhCu/TiO_2_. Cu^0^ and Cu^+^ are difficult to distinguish because they appeared at almost the same position in XPS spectra. Therefore, even if electrons are transferred from Cu to Rh, we cannot observe a clear change in the Cu 2p XPS spectra because electron-poor Cu^0^ appears at an almost similar position to pure Cu^0^. And, we roughly estimated the ratio of the peak derived from single Rh metal and the RhCu alloy by fitting. The ratio of the peak derived from single Rh metal and the RhCu alloy is estimated to be 42.2% and 57.8%, respectively.

**Fig. 2:**
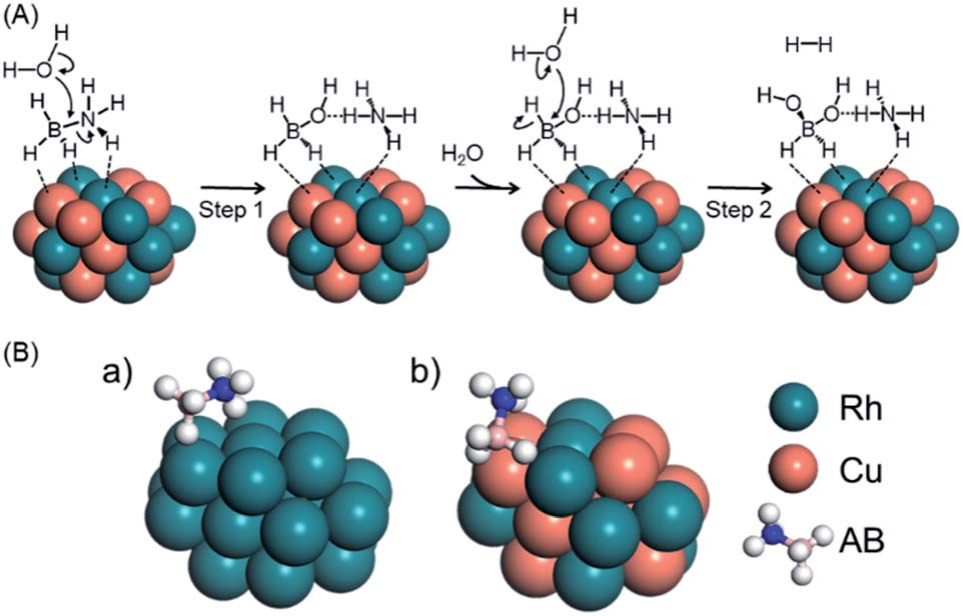
(A) Schematics showing possible processes for AB hydrolysis and (B) the calculated model for the adsorption of AB on (a) Rh24 or (b) Rh12Cu12 clusters with the (111) facet exposed.

Previous experimental and theoretical studies reported the possible mechanism for AB hydrolysis using metal NPs. Feng *et al.* observed changes of the Cu state during AB hydrolysis using *in situ* XAFS, which showed that AB molecules interact with the catalyst firstly and water can approach both the catalyst and AB to attack the N–B bond.^61^ Peng *et al.* demonstrated the plausible AB hydrolysis mechanism using DFT calculations.^57^ Thus, we show the possible mechanism of AB hydrolysis in Fig. 2A, and the details are as follows. In this cycle, the reaction is initiated by the dissociation of the N–B bond in AB, followed by the attack of a H_2_O molecule to generate BH_3_(OH)^−^ and NH_4_^+^. Subsequently, hydrogen is produced by a second attack of H_2_O on the BH_3_(OH)^−^ ion. Finally, two hydrogen molecules are released from BH_2_(OH)_2_^−^ accompanied by the formation of BO_2_^−^. It should be noted that the final step proceeds readily because the associated energy barrier is lower than those for the other steps. Based on this mechanism, we estimated the activation energy (*E*_a_) values for the first and second steps for Rh_24_ and Rh_12_Cu_12_ clusters (Fig. 2A, Table 1). *E*_a_ for the first step was determined to be 24.8 kcal mol^−1^ on the Rh_12_Cu_12_, which is much lower than that on the Rh cluster, for which *E*_a_ was 38.5 kcal mol^−1^. In addition, the *E*_a_ values for the second step were lower than those for the first step for both the Rh and RhCu clusters. These results demonstrate that the initial attack by H_2_O is the rate determining step during this reaction, and is promoted by the presence of Rh_12_Cu_12_ clusters having electron-rich Rh species. Calculated models in the rate-limiting step are shown in Fig. S7.† It is well known that a greater adsorption energy value (*E*_ad_) for the reaction intermediate on the metal catalyst corresponds to a lower reaction barrier, according to the Brønsted–Evans–Polanyi (BEP) relationship.^62^ The *E*_ad_ for adsorption on the Rh_12_Cu_12_ cluster was calculated to be 22.1 kcal mol^−1^, which is 1.6 times the value of 14.1 kcal mol^−1^ for the Rh_24_ cluster (Fig. 2B, Table 1). Thus, sites comprising neighboring Rh–Cu pairs with an electronic imbalance evidently act to promote interactions with AB molecules, enhancing the catalytic activity.

**Table 1:** (A) Activation energy for AB hydrolysis in the reaction pathway in Fig. 2 and (B) adsorption energy of AB on (a) Rh_24_ or (b) Rh_12_Cu_12_ clusters

	Clusters	(A) Activation Energy		(B) Adsorption Energy of AB/kcal mol^−1^
		First attack of H_2_O (step 1)/kcal mol^−1^	Second attack of H_2_O (step 2)/kcal mol^−1^	
a	Rh_24_	38.5	10.4	14.1
b	Rh_12_Cu_12_	24.8	15.0	22.1

### Characterization of RhCu/TiO_2_

**Fig. 3:**
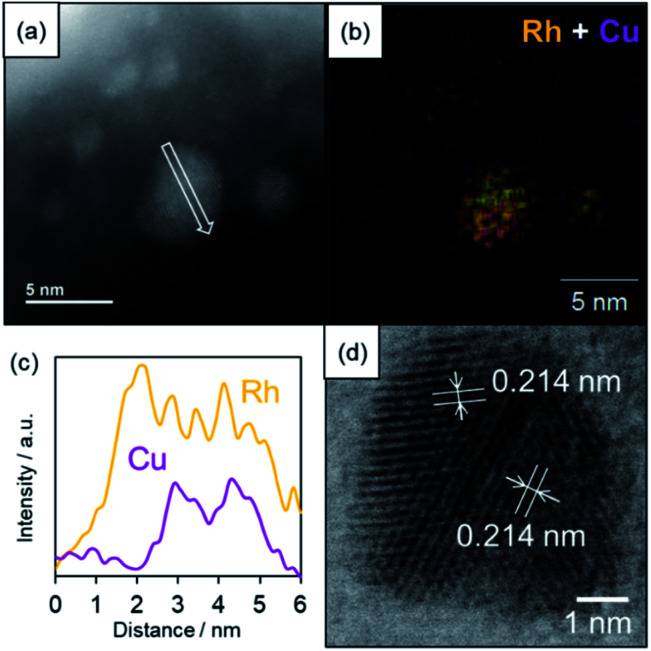
HAADF-STEM and BF-STEM images and EDX analysis of the RhCu/TiO_2_. (a) A HAADF-STEM image, (b) an EDX map of Rh + Cu in the region indicated in (a), (c) EDX line analysis along the arrow in (a), and (d) a BF-STEM image showing the lattice distances.

The formation of a solid solution RhCu alloy on the TiO_2_ support was confirmed by various characterization experiments. Fig. 3 presents a high-angle annular dark field scanning TEM (HAADF-STEM) image, a bright field STEM (BF-STEM) image, energy dispersive X-ray (EDX) maps, and a line analysis for a RhCu/TiO_2_ catalyst after reduction at 350 °C under a H_2_ atmosphere. The average size of the RhCu NPs was estimated to be 2.0 nm based on the STEM image, while the EDX maps confirm the formation of a RhCu alloy, since both Rh and Cu signals appeared in the same region. The EDX line analysis shows both Rh and Cu signals in the same area, demonstrating the presence of a solid solution RhCu alloy. Fig S8† presents EDX line analysis of smaller RhCu NPs at around 2–3 nm. The EDX line analysis showed that this nanostructure was found to be a solid solution structure since the Rh and Cu signal appeared at a similar position. However, the atomic ratio of Rh : Cu is 68 : 32 in this particle from the X-ray intensity of Rh and Cu, indicating the formation of the Rh-rich RhCu alloy even in smaller particles. Lattice fringes are evident in the BF-STEM image of the RhCu NPs and the corresponding *d* spacing was estimated to be 0.214 nm in various regions. This value is almost equal to the average of the spacings of the Rh (111) planes (0.219 nm) and the Cu (111) planes (0.207 nm). These findings again confirm that Rh and Cu form a solid solution structure on the TiO_2_.

The reduction temperatures of the supported metal precursors were determined using H_2_-TPR (Fig. S9†). Typically, a Rh precursor will be reduced faster than a Cu precursor because the reduction potential of Rh^3+^ is more positive than that of Cu^2+^ (Cu^2+^ + 2e^−^ → Cu : *E*_0_ = 0.34 *vs.* RHE, Rh^3+^ + 3e^−^ → Rh : *E*_0_ = 0.76 *vs.* RHE) although both ions show a positive reduction potential. The reductions of Rh^3+^ species in the Rh/γ-Al_2_O_3_, Rh/MgO, and Rh/TiO_2_ were found to occur at around 70, 120, and 150 °C, respectively. In contrast, the reductions of Cu^2+^ species in the Cu/γ-Al_2_O_3_ and Cu/MgO were observed to proceed above 300 °C, while reduction in the Cu/TiO_2_ occurred at a significantly lower temperature of approximately 240 °C. Interestingly, the RhCu/TiO_2_ exhibited two-step reduction peaks approximately at 140 and 210 °C. It is considered that the peak at lower temperature (around 140 °C) represents reduction of some of the Rh^3+^ to Rh^0^ (Rh nuclei) and the peak at higher temperature (around 210 °C) represents simultaneous reduction of other Rh^3+^ and Cu^2+^. This result indicates that the Rh^3+^ and Cu^2+^ precursors were reduced simultaneously on the TiO_2_ support to produce a solid solution alloy. A similar trend was also observed on the TiO_2_ (A). In contrast, the RhCu/γ-Al_2_O_3_ and RhCu/MgO specimens produced two reduction peaks, suggesting the individual reductions of Rh^3+^ and Cu^2+^ precursors.

**Fig. 4:**
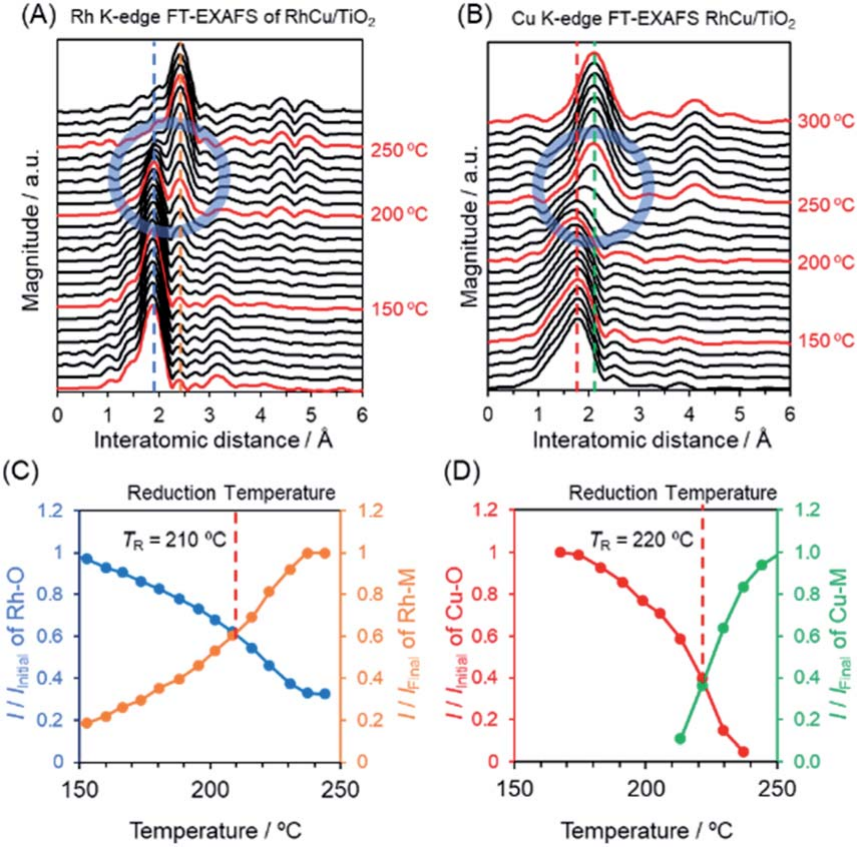
*In situ* FT-EXAFS spectra of the (A) Rh K-edge of RhCu/TiO_2_ and (B) Cu K-edge of RhCu/TiO_2_ acquired during reduction under H_2_ at elevated temperature. Variations in the intensities of peaks attributed to (C) Rh–O and Rh-M (metal) bonds based on the data in (A) and (D) Cu–O and Cu-M bonds based on the data shown in (B). (C and D) Vertical axis shows relative intensity compared to that of initial data (before H_2_ reduction) attributed to the Rh–O bond and compared to that of final data (after H_2_ reduction) attributed to the Rh–Metal(M) bond.

To further elucidate changes in the local structures of the Rh, Cu and RhCu supported catalysts, *in situ* XAFS analyses were performed during a reduction sequence under H_2_ at elevated temperatures (Fig. S10–S15†). The Rh K-edge FT-EXAFS spectrum produced by the RhCu/TiO_2_ (Fig. 4A) exhibits a sharp singlet attributed to Rh–O bonds at approximately 1.9 Å below 200 °C, whereas another peak attributed to metallic Rh bonds appeared at approximately 2.4 Å above 200 °C. This transition in the FT-EXAFS spectra demonstrates the reduction of Rh^3+^ on the TiO_2_ support. Fig. 4C summarizes changes in the intensities of the peaks ascribed to Rh–O and Rh–Rh(Cu) bonds during heating of the RhCu/TiO_2_. The Cu K-edge *in situ* FT-EXAFS spectra of RhCu/TiO_2_ and the changes in the intensities of peaks due to Cu–O and Cu–Cu(Rh) bonds during heating are shown in Fig. 4B and D. Similarly, Fig. S10–S15† present the *in situ* XAFS spectra acquired from Rh, Cu and RhCu supported on TiO_2_, γ-Al_2_O_3_ and MgO and the transition data for all specimens are shown in Fig. S16–S18.† The intersection between the transitions of two peaks was defined as the reduction temperature (*T*_R_) for each metal precursor on the various supports, and these values are summarized in Table 2.

**Table 2:** Reduction temperatures of Rh^3+^ and Cu^2+^ supported on TiO_2_, γ-Al_2_O_3_ and MgO as determined based on *in situ* FT-EXAFS spectra

	Rh K-edge	Cu K-edge
Rh/TiO_2_	200 °C	
Cu/TiO_2_		280 °C
RhCu/TiO_2_	210 °C	220 °C
Rh/γ-Al_2_O_3_	60 °C	
Cu/γ-Al_2_O_3_		310 °C
RhCu/γ-Al_2_O_3_	130 °C	300 °C
Rh/MgO	130 °C	
Cu/MgO		310 °C
RhCu/MgO	130 °C	310 °C

The *T*_R_ for Rh^3+^ on the Rh/TiO_2_ was 200 °C and this value was slightly increased to 210 °C in the case of the RhCu/TiO_2_. As expected, the *T*_R_ for Cu^2+^ on the Cu/TiO_2_ was drastically decreased (from 280 to 220 °C) after mixing with Rh^3+^. The *T*_R_ values for Rh^3+^ and Cu^2+^ in the RhCu/TiO_2_ were intermediate between those corresponding to the single Rh and Cu samples, which suggests that the two precursors were simultaneously reduced on the TiO_2_ surface. However, the *T*_R_ values for Cu^2+^ on the γ-Al_2_O_3_ and MgO supports were above 300 °C, and remained high even after mixing with Rh^3+^. These observations indicate that neither the Rh nor the Cu precursor significantly affects the reduction profile of the other, and that the reductions of these two metals occur individually on the γ-Al_2_O_3_ and MgO surfaces. In these cases, segregated monometallic NPs and/or Rh_core_–Cu_shell_-type NPs are presumably obtained as opposed to the binary alloy.

It is unlikely that Cu^2+^ will be reduced as fast as reduction of Rh^3+^ on the TiO_2_ support because the reduction potential of Cu is lower than that of Rh. Based on the above results, it is probable that the reductions of Rh^3+^ and Cu^2+^ species on the TiO_2_ support proceed *via* a unique mechanism compared to the mechanisms on the other supports. A mechanism by which Rh^3+^ and Cu^2+^ are reduced on the TiO_2_ support can be proposed by considering the contribution of hydrogen spillover from the Rh to the Cu.^30^ Hydrogen spillover is known to promote the reduction of metals on various supports through hydrogen dissociation on a second precious metal.^31,32^ During the initial stage of the reduction, it is thought that pre-reduced Rh nuclei dissociate hydrogen molecules, after which the hydrogen is transferred to the Cu^2+^ precursor by spillover such that the ions are reduced to Cu^0^. Metal oxide supports with reversible reducibility, such as TiO_2_, also tend to promote spillover ability.^33^

**Fig. 5:**
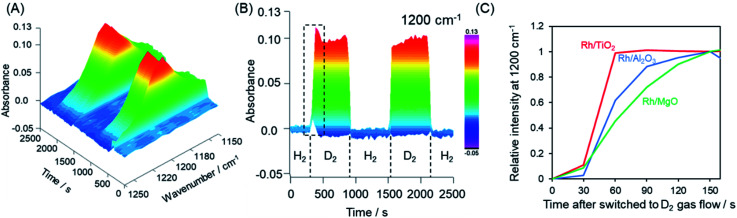
(A) 3D and (B) 2D time courses of the changes in the intensity of the peak at 1200 cm^−1^ attributed to the *δ*_D–O–D_ stretching vibration as obtained from *in situ* FTIR spectra acquired during the H_2_ and *D*_2_ gas exchange sequence over Rh/TiO_2_. (C) Normalized peak intensities after *D*_2_ gas introduction within the area indicated by dashed lines in (B) for Rh/TiO_2_, Rh/Al_2_O_3_ and Rh/MgO.

The hydrogen spillover characteristics of the TiO_2_, γ-Al_2_O_3_, and MgO supports were compared by acquiring *in situ* FTIR spectra during sequential H_2_ and *D*_2_ gas flows over Rh supported catalysts. If rapid hydrogen spillover occurred on a support, we would expect to observe rapid exchange between gas phase hydrogen and support protons *via* the spillover process.^54^ Initially, physisorbed water was removed from the pre-reduced samples over the span of 1 h, after which no further changes in the spectrum were observed. Subsequently, the sample was placed under a H_2_ atmosphere and heated at 50 °C, and then allowed to equilibrate. Following this, the gas flow was switched to *D*_2_ instead of H_2_ and then back to H_2_. This gas exchange procedure was repeated, and the 3D changes over time in the 1200 cm^−1^ peak (attributed to the *δ*_D–O–D_ stretching vibration) are shown in Fig. 5A–C and S19.†Fig. 5C displays the normalized intensity of the 1200 cm^−1^ peak for Rh supported on TiO_2_, γ-Al_2_O_3_, and MgO supports. After switching from H_2_ to *D*_2_, the Rh/MgO required more than 150 s to saturate, while the Rh/γ-Al_2_O_3_ saturated at approximately 150 s. It should be noted that the increase in the rise in the peak intensity during trials with the Rh/TiO_2_ was complete within only 60 s. The process required 30 s for each observation to integrate the data and another several seconds to replace the gases after switching from H_2_ to *D*_2_. Thus, the rate of peak saturation for the Rh/TiO_2_ was too fast and almost fell below the resolution limitation (30 s) of the *in situ* FT-IR system after the *D*_2_ gas reached the catalyst. Based on the H-D exchange kinetics, we can conclude that rates of hydrogen spillover on the Rh supported TiO_2_ specimen were much higher than thsoe for Rh on non-reducible supports such as γ-Al_2_O_3_ and MgO.

**Fig. 6:**
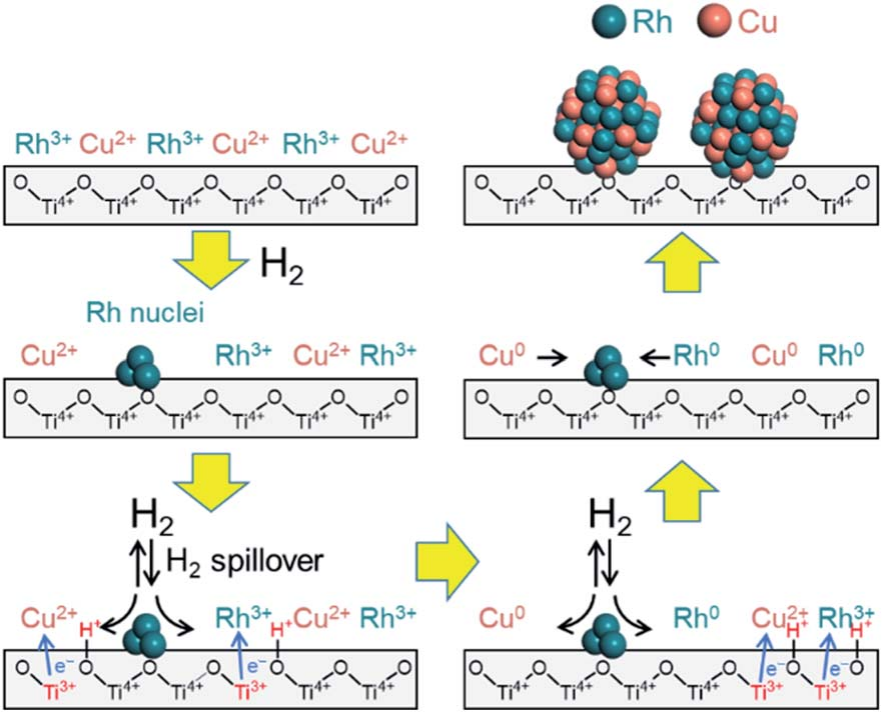
A proposed mechanism for the formation of an immiscible RhCu binary alloy on a TiO_2_ support assisted by hydrogen spillover.

Considering these results, we propose a mechanism for the formation of the solid solution RhCu alloy on the TiO_2_ support (Fig. 6). During these trials, the sample was heated under a H_2_ atmosphere such that Rh^3+^ was partially reduced to generate Rh metal nuclei at around 140 °C. The nuclei seem to be formed at the defect site of TiO_2_ and hardly involved in alloy formation since they are stable. Following this, hydrogen was dissociated on the surfaces of these Rh nuclei to form Rh–H species, accompanied by the reduction of Ti^4+^ to Ti^3+^ at the metal–support interfaces. Electrons migrated from Ti^3+^ ions to neighboring Ti^4+^ ions, which allowed simultaneous proton transfer to O^2−^ anions attached to adjacent Ti^4+^. In this manner, the hydrogen rapidly reached both metal ions (Rh^3+^ and Cu^2+^) by moving over the TiO_2_ surface, and these ions were reduced at the same time to form RhCu clusters. These clusters then aggregated to produce the non-equilibrium RhCu alloy. This proposed mechanism is supported by the synergistic effect that evidently promotes the catalytic activity of the RhCu/TiO_2_ compared to the Rh/TiO_2_ following reduction at various temperatures (Fig. S20†). The rate of increase in catalytic activity was found to be significantly dependent on the reduction temperature and increased in the following order: 200 °C (1.38) < 250 °C (2.33) < 300 °C (2.55) < 350 °C (2.60). The decrease of TOF over Rh/TiO_2_ and RhCu/TiO_2_ catalysts showed an almost similar rate at reduction temperature over 250 °C, indicating that TOF decreases are related to the particle size. Moreover, RhCu/TiO_2_ is considered to show a promotional alloying effect at reduction temperature over 220 °C, and in fact, this catalyst suddenly showed more than 2.3 higher activity compared to the Rh/TiO_2_ catalyst at reduction temperature over 250 °C, indicating that the activity improvement is due to the formation of the solid solution alloy. This result is consistent with the data obtained by H_2_-TPR and *in situ* XAFS analyses. In addition, the BET surface area of supports was measured to be 100, 338, 185, 11.3 and 2.53 m^2^ g^−1^ for TiO_2_ rutile, TiO_2_ anatase, γ-Al_2_O_3_, ZrO_2_ and MgO, respectively. This result showed even if γ-Al_2_O_3_ which has a higher surface area than TiO_2_ rutile is used, the corresponding alloy was not formed. Hence, it can be concluded that the participation of spillover is more important than the distribution of Rh and Cu on the support, for solid solution alloy formation with immiscible Rh and Cu combination.

**Fig. 7:**
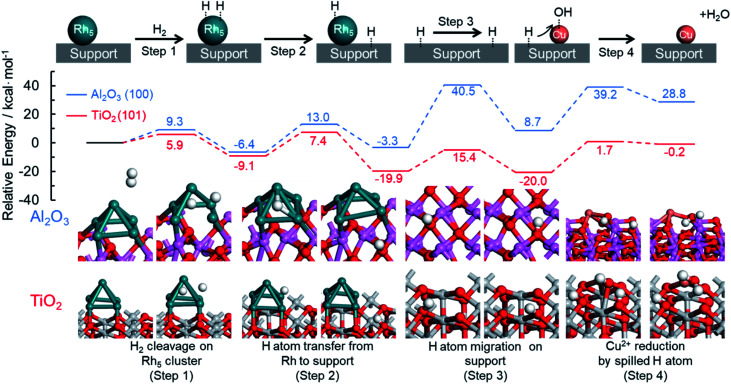
Theoretical pathway for the reduction of Cu species on the TiO_2_ and Al_2_O_3_ supports assisted by hydrogen spillover.

DFT calculations were also performed to provide further support for this mechanism by which an immiscible alloy is produced based on the spillover effect. The resulting potential energy profiles are summarized in Fig. 7. Rutile TiO_2_ (101) and hexagonal Al_2_O_3_ (100) planes were chosen as models for this theoretical investigation because of their thermodynamic stability, and Rh_5_ clusters were employed as a model of Rh nuclei. Four representative reaction steps were considered for the reduction of Cu^2+^ species on the support through the hydrogen spillover from Rh clusters, according to the above proposed reaction mechanism. These steps included the dissociation of H_2_ on a Rh cluster loaded on the support (step 1), the transfer of a dissociated H atom from a Rh cluster to a neighboring O atom on the support (step 2), the migration of a H atom over the support surface (step 3), and the reduction of Cu^2+^ by the spilled H atom on the support (step 4).

In the case of TiO_2_ (101), the dissociation of H_2_ on a Rh_5_ cluster (step 1) occurs with a barrier of 5.9 kcal mol^−1^ (Fig. S21†). The *E*_a_ associated with H atom transfer from a Rh_5_ cluster to a neighboring O atom on the support (step 2) was estimated to be 16.5 kcal mol^−1^ (Fig. S22†). These values are similar to those obtained using Al_2_O_3_ (100), for which the *E*_a_ values were 9.3 and 19.4 kcal mol^−1^, respectively. The migration of a H atom over the support surface (step 3) is considered a separate step because of the presence of oxygen sites having different coordination numbers, such as 2-coordinated oxygen (O(2)) or 3-coordinated oxygen (O(3)) sites. The barriers for H atom migration from O(2) to O(2), O(2) to O(3), and O(3) to O(2) were calculated to be 15.0, 37.4, and 12.7 kcal mol^−1^ on the TiO_2_ (101), respectively. These results demonstrate that H atom migration over the TiO_2_(101) preferentially occurs at O(2) sites because TiO_2_(101) has numerous O(2) sites and because the participation of O(3) is energetically unfavorable. In the case of Al_2_O_3_(100), the *E*_a_ values were determined to be 29.3, 41.7, and 43.8 kcal mol^−1^ for the transfer pathways from O(2) to O(3), O(3) to O(2), and O(3) to O(3), respectively (Fig. S23†). Since the number of O(2) sites on Al_2_O_3_ is small, H atom migration on this substrate preferentially occurs at the O(3) sites, and even if an O(2) site is adjacent to O(3) sites, there is a relatively high activation energy for migration from O(2) to O(3) sites. These results clearly suggest that H atom transfer on TiO_2_ is energetically more likely to proceed than that on Al_2_O_3_, and hence the rate of hydrogen spillover is faster on the TiO_2_.

With respect to the reduction of Cu^2+^ by the spilled H atom on the support (step 4), we calculated the activation energy for the attack of a neighboring H atom on a Cu–OH group on the support, followed by the loss of H_2_O. The *E*_a_ values were estimated to be 21.7 and 30.5 kcal mol^−1^ on the TiO_2_(101) and Al_2_O_3_(100), respectively (Fig. S24†). Taking into account the overall reactions, the reduction of Cu^2+^ species on TiO_2_ proceeds with a barrier of 21.7 kcal mol^−1^ in the presence of Rh, while that on Al_2_O_3_ requires an activation energy of 43.8 kcal mol^−1^ even in the presence of Rh. The energy of the final state on the Al_2_O_3_ appeared to be thermodynamically high since all the steps for reduction of Cu^2+^ with hydrogen spillover from Rh are described together in Fig. 7. In detail, step 2 and step 3 are thermodynamically unfavorable steps which are related to hydrogen migration to the Al_2_O_3_ surface. Moreover, the same calculations were also performed on the γ-Al_2_O_3_ model reported by Digne *et al.*, since their structure has been widely accepted for the investigations.^63,64^ In the theoretical reduction pathway, H atom migration on the support has the highest activation energy (Fig. S27, Table S1†). The obtained activation energy for the hydrogen migration (38.9 kcal mol^−1^) is almost similar to a value of our result on hexagonal Al_2_O_3_ (41.7–43.8 kcal mol^−1^). In addition, Bokhoven *et al.* calculated the activation energy of hydrogen spillover on γ-Al_2_O_3_ using the model reported by Digne.^33^ The corresponding value (37.6 kcal mol^−1^) is the same as our results, indicating the reliability of our calculations. These results produced by the theoretical investigations help to explain the differences in the reduction temperatures for Cu^2+^ species between the RhCu/TiO_2_ and RhCu/Al_2_O_3_. In preliminary work, the energy changes associated with the direct reduction of Cu^2+^ by H_2_ molecules on each support were calculated, giving values of 36.8 and 44.7 kcal mol^−1^ on the TiO_2_(101) and Al_2_O_3_(100), respectively (Fig. S25†). These values correspond to the reduction of Cu/TiO_2_ and Cu/Al_2_O_3_, respectively. The dissociation energies of gaseous H_2_ on TiO_2_(101) and Al_2_O_3_(100) without Rh clusters were estimated to be greater than 80 kcal mol^−1^ (Fig. S26†), and so the reduction of Cu^2+^ must proceed solely due to spilled H atoms in the presence of Rh. These calculated *E*_a_ values and the experimental Cu^2+^ reduction temperatures on the TiO_2_ and γ-Al_2_O_3_ determined by *in situ* XAFS are summarized in Table 3. On the TiO_2_ support, the reduction of Cu^2+^ species occurs with an *E*_a_ of 21.7 kcal mol^−1^ in the presence of Rh (210 °C) and with an *E*_a_ of 36.8 kcal mol^−1^ in the absence of Rh (280 °C). On the Al_2_O_3_ support, these values are 43.8 kcal mol^−1^ with Rh (300 °C) and 44.7 kcal mol^−1^ without (310 °C). These correlations between *E*_a_ and the experimental reduction temperature are in good agreement with the proposed mechanism for the formation of the solid solution RhCu alloy.

**Table 3:** (A) Activation energies for various steps during the reduction of Cu^2+^ species assisted by hydrogen spillover on TiO_2_ and Al_2_O_3_ and (B) experimental temperatures for the reduction of Cu^2+^ on the catalyst as determined from *in situ* XAFS data

	(A) Activation Energy/kcal mol^−1^				(B) Reduction Temperature of Cu^2+^/°C
	H_2_ cleavage on the Rh_5_ cluster	H atom transfer from the Rh_5_ cluster to the support	H atom migration on the support	Cu^2+^ reduction by spilled H atoms	
TiO_2_ (101)	5.9	16.5	15.0	21.7	210 (RhCu/TiO_2_)
Al_2_O_3_ (100)	9.3	19.4	41.7–43.8	30.5	300 (RhCu/γ-Al_2_O_3_)
	H_2_ cleavage on the support			Cu–O reduction by H_2_ vapor	Reduction temperature/°C
TiO_2_ (101)	82.3			36.8	280 (Cu/TiO_2_)
Al_2_O_3_ (100)	89.3			44.7	310 (Cu/γ-Al_2_O_3_)

The phase diagram for Rh and Cu shows that these metals are essentially immiscible at most compositions and do not form solid solution alloys below 600 °C, because of the positive enthalpy of formation of such alloys. Thus, the synthesis of such alloys is difficult using conventional methods. In addition, γ-Al_2_O_3_, MgO, and ZrO_2_ supports do not afford RhCu alloys under the present synthetic conditions, indicating that the use of TiO_2_ as a platform for the synthesis of alloy NPs could be a useful means of obtaining solid solution alloy structures with immiscible combinations.

## Conclusions

TiO_2_ has been demonstrated to be an efficient platform for the formation of a RhCu binary alloy with immiscible combination under H_2_ reduction conditions. A RhCu/TiO_2_ catalyst synthesized in this manner showed 2.6 times higher activity than Rh/TiO_2_ during hydrogen production from the hydrolysis of AB. This activity improvement was not obtained when using other supports. Theoretical studies revealed that the adsorption energy of AB was increased on the RhCu binary alloy, whereas the activation energy for dissociation of the N–B bond (the rate-determining step) was decreased compared to the value obtained using monometallic Rh. This change in the energy barrier is attributed to the formation of negatively charged Rh atoms and positively charged Cu atoms in close proximity to one another. The generation of a RhCu binary alloy on the TiO_2_ support was confirmed by HAADF-STEM and EDX observations. Reduction profiles obtained using H_2_-TPR and *in situ* XAFS data suggest the simultaneous reduction of Rh^3+^ and Cu^2+^ species on the TiO_2_ support. The rapid H_2_/D_2_ exchange reaction observed by *in situ* FT-IR spectroscopy suggests that the rate of hydrogen spillover increased on TiO_2_. From these results, we conclude that Rh^3+^ ions are first reduced by H_2_ to generate nuclei, after which the dissociation of hydrogen molecules occurs to form hydride species that act as cocatalysts to reduce neighboring Cu^2+^ species. This process is assisted by the hydrogen spillover characteristics of the TiO_2_*via* a coupled proton–electron transfer mechanism. Theoretical simulations provide evidence for the facilitated migration of H atoms on the TiO_2_ and show that the spilled H atoms enable the reduction of Cu^2+^ species with a lower activation energy compared to direct H_2_ reduction. This study demonstrates a promising approach for the preparation of non-equilibrium solid solution alloys, and we expect that this strategy will be extendable to other combinations of metals that may serve as unique catalysts and/or photocatalysts.

## Conflicts of interest

There are no conflicts to declare.

## Supplementary Material

SC-011-C9SC05612B-s001
