# Supplementary material for: Synthesis of a binary alloy nanoparticle catalyst with an immiscible combination of Rh and Cu assisted by hydrogen spillover on a TiO2 support
Source: Chem Sci. 2020 Apr 1;11(16):4194–203. doi: 10.1039/c9sc05612b (PMC8152661; doi:10.1039/c9sc05612b)
Supplement: SC-011-C9SC05612B-s001 [file SC-011-C9SC05612B-s001.pdf]

Electronic Supplementary Material (ESI) for Chemical Science.
This journal is © The Royal Society of Chemistry 2020

Supporting Information

Synthesis of an Immiscible RhCu Bimetallic Alloy Nanoparticle Catalyst Assisted by Hydrogen Spillover on a TiO_2 Support

Shinya Masuda,[a] Kazuki Shun,[a] Kohsuke Mori,*[a,b] Yasutaka Kuwahara[a,b] and Hiromi Yamashita*[a,b]

[a]Division of Materials and Manufacturing Science, Graduate School of Engineering, Osaka University, 2-1 Yamadaoka, Suita, Osaka 565-0871, Japan
[b]Unit of Elements Strategy Initiative for Catalysts Batteries (ESICB), Kyoto University, Katsura, Kyoto 615-8520, Japan

Corresponding Author
*K.M.: tel and fax, +81-6-6879-7460; e-mail, mori@mat.eng. osaka-u.ac.jp.
*H.Y.: tel and fax, +81-6-6879-7457; e-mail, yamashita@mat. eng.osaka-u.ac.jp.

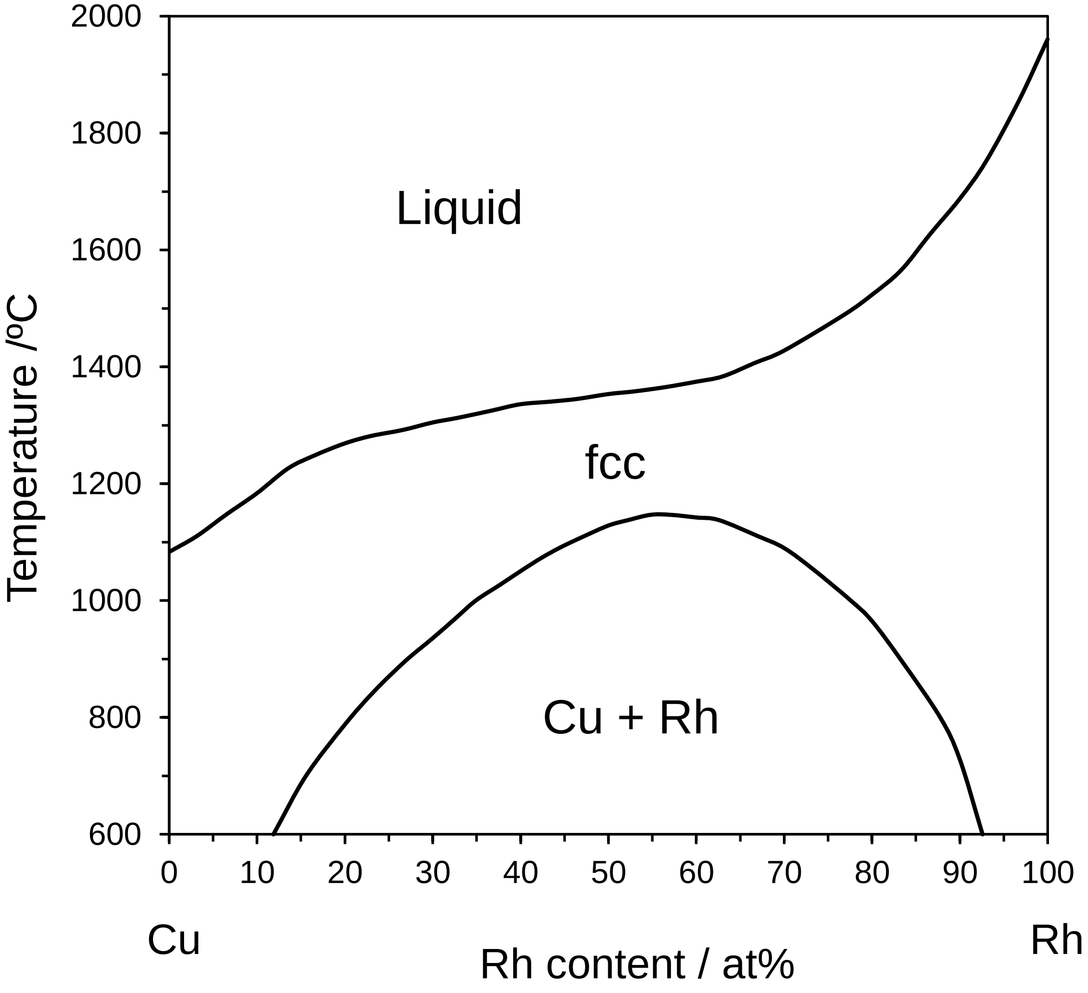

Fig. S1 Phase diagram of Rh and Cu.

Chakrabarti, D. J.; Laughlin, D. E., Cu−Rh. *Journal of Phase Equilibria* **1982**, *2*, 511-512.

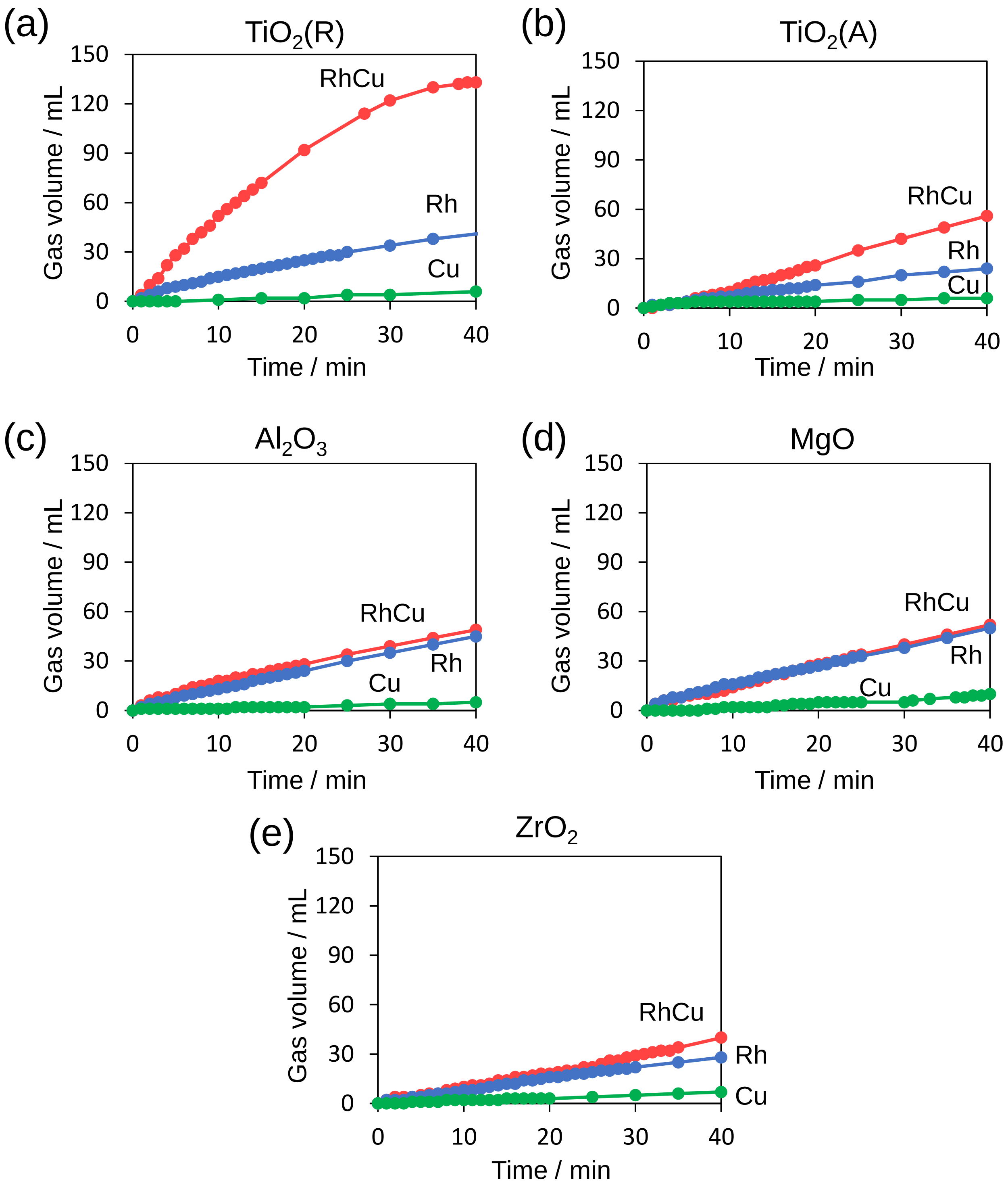

Fig. S2 Time course in the hydrogen production from the AB hydrolysis over Rh, Cu and RhCu supported catalysts on (a) TiO_2 (R), (b) TiO_2 (A), (c) Al_2O_3, (d) MgO and (e) ZrO_2. Catalytic conditions; catalyst 20 mg, 10 mL of AB aqueous solution (0.2 M) at 323 K.

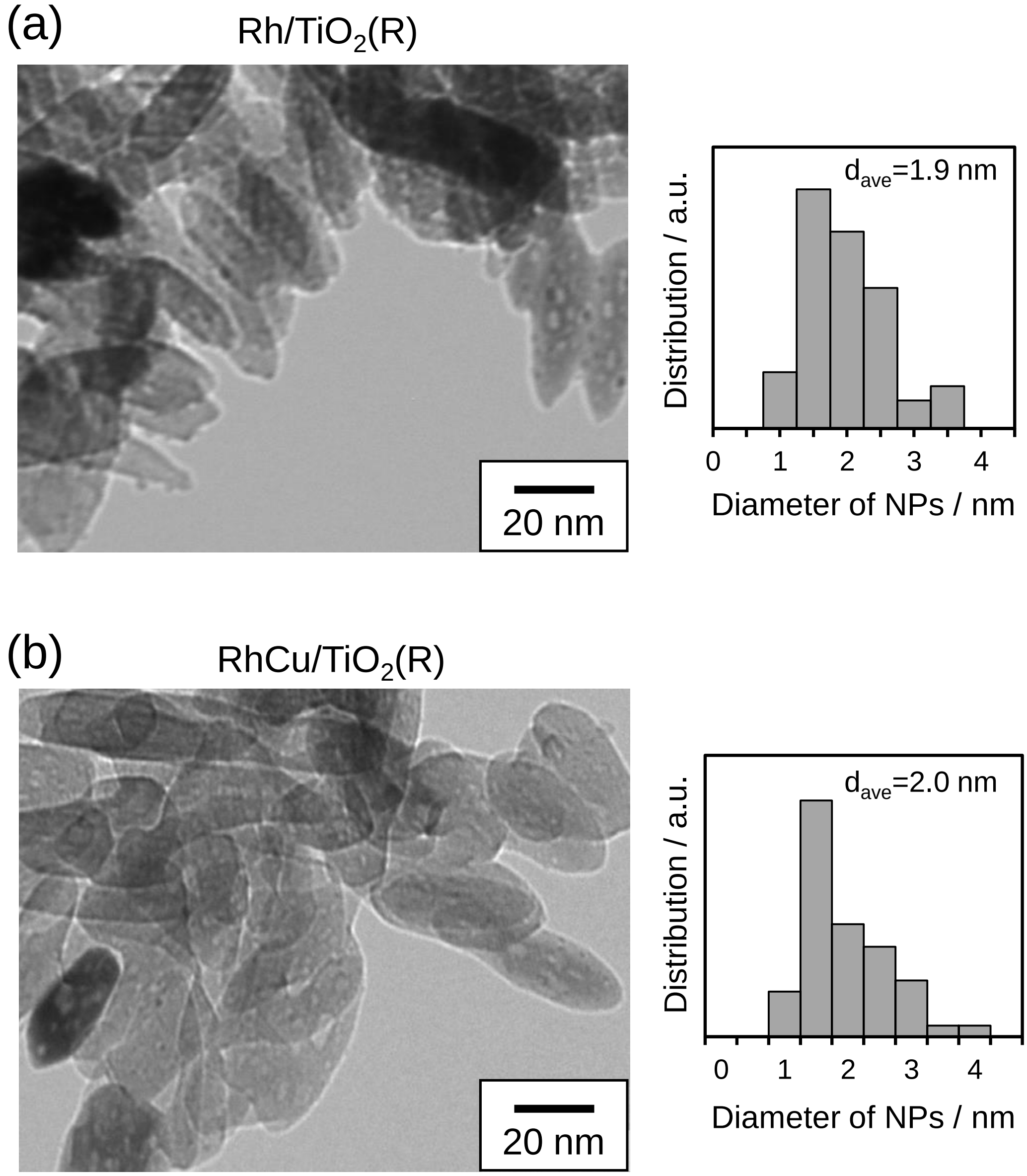

Fig. S3 TEM image and size distribution of Rh or RhCu NPs of the (a) Rh/TiO_2 and (b) $RhCu/TiO_2$ catalysts.

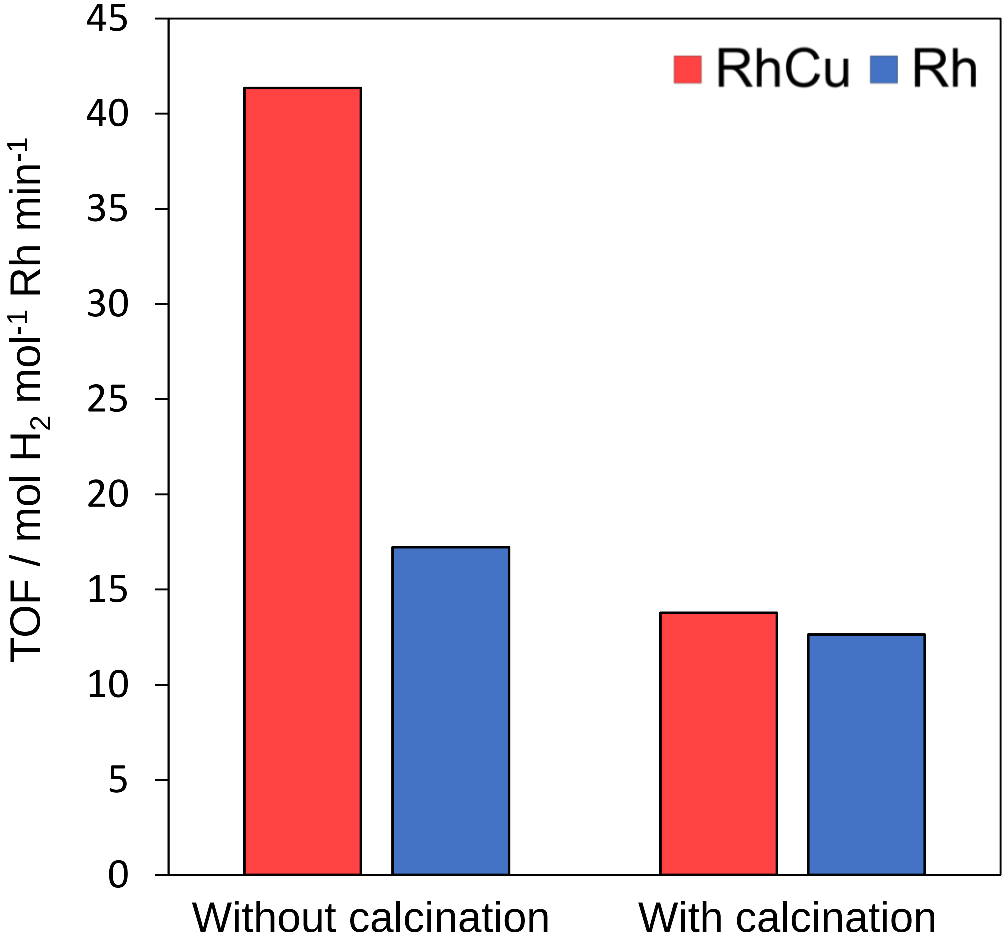

Fig. S4 Pre-calcination effect of Rh and RhCu supported TiO_2 catalysts before hydrogen reduction toward AB hydrolysis. Treatment conditions: calcined at 500 ºC for 3h and reduced at 350 ºC for 2 h under H_2 atmosphere (catalytic conditions: catalyst 20 mg, 10 mL 0.2 M aqueous AB solution, 30 ºC).

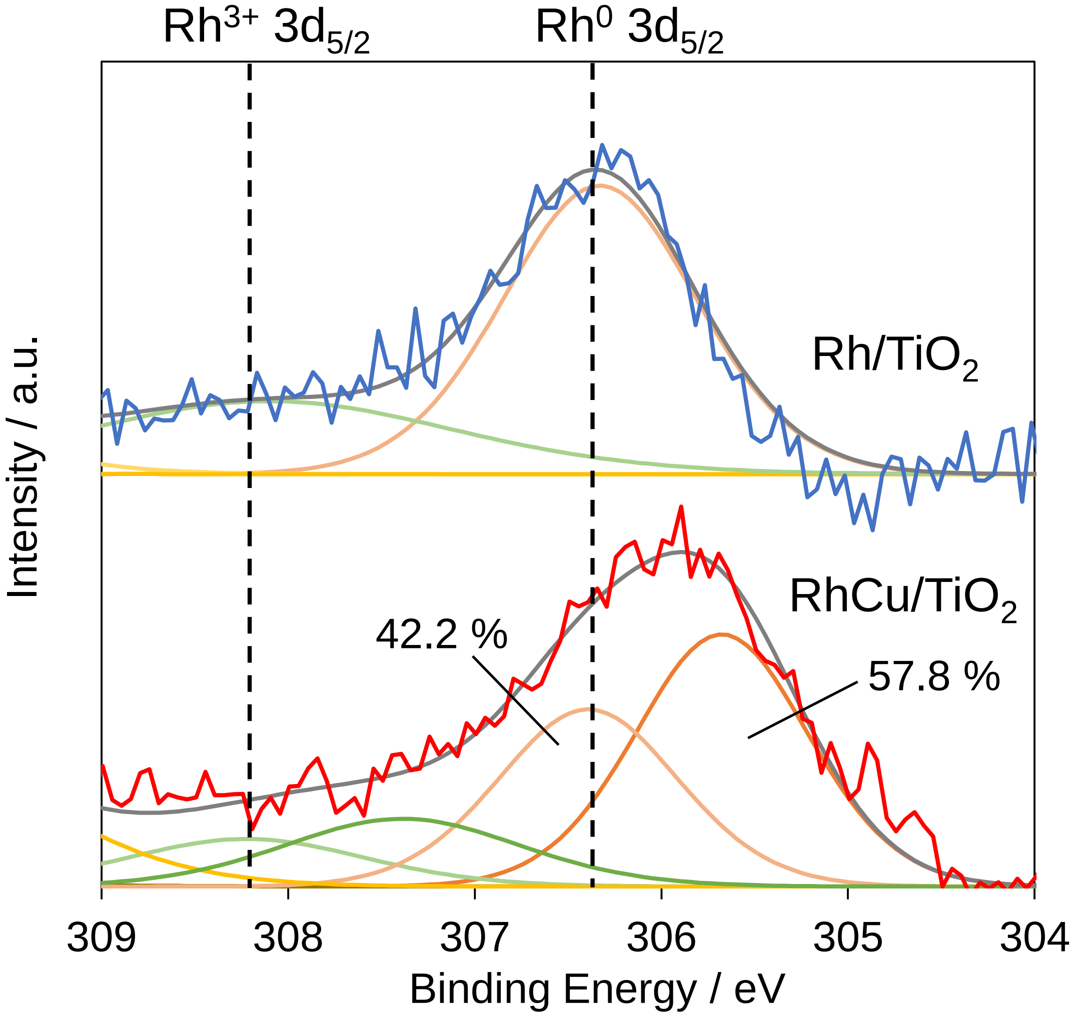

Fig. S5 Rh 3d XPS spectra of Rh/TiO_2 and RhCu/TiO_2 catalysts.

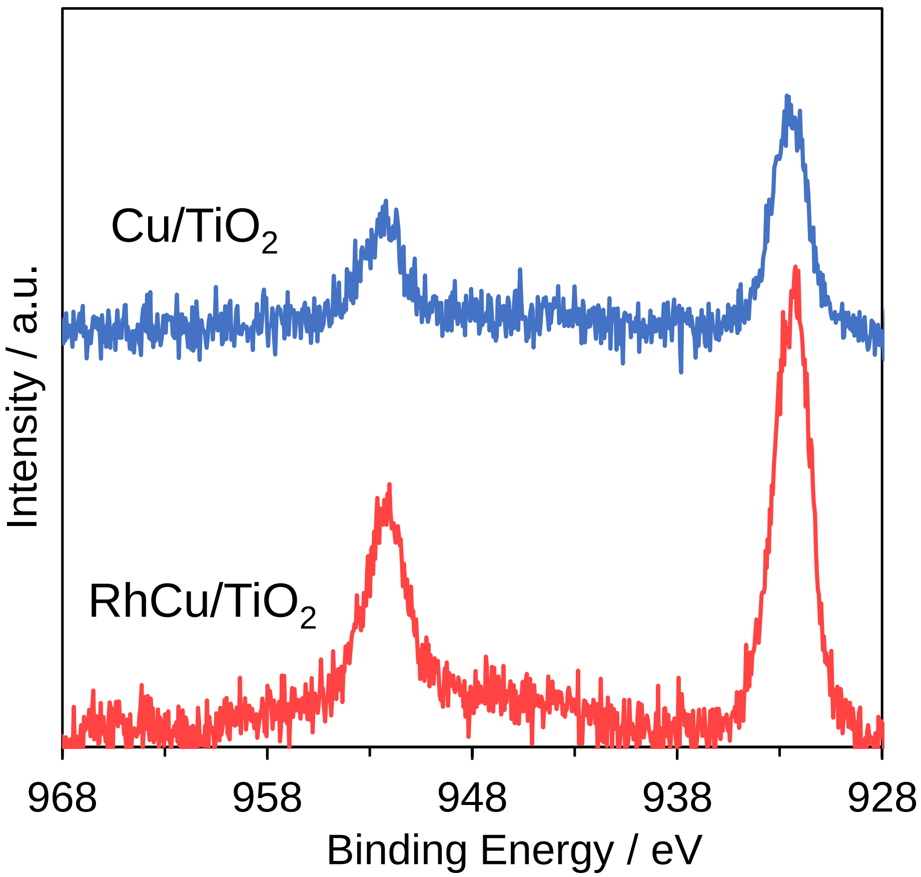

Fig. S6 Cu 2p XPS spectra of Cu/TiO_2 and RhCu/TiO_2 catalysts.

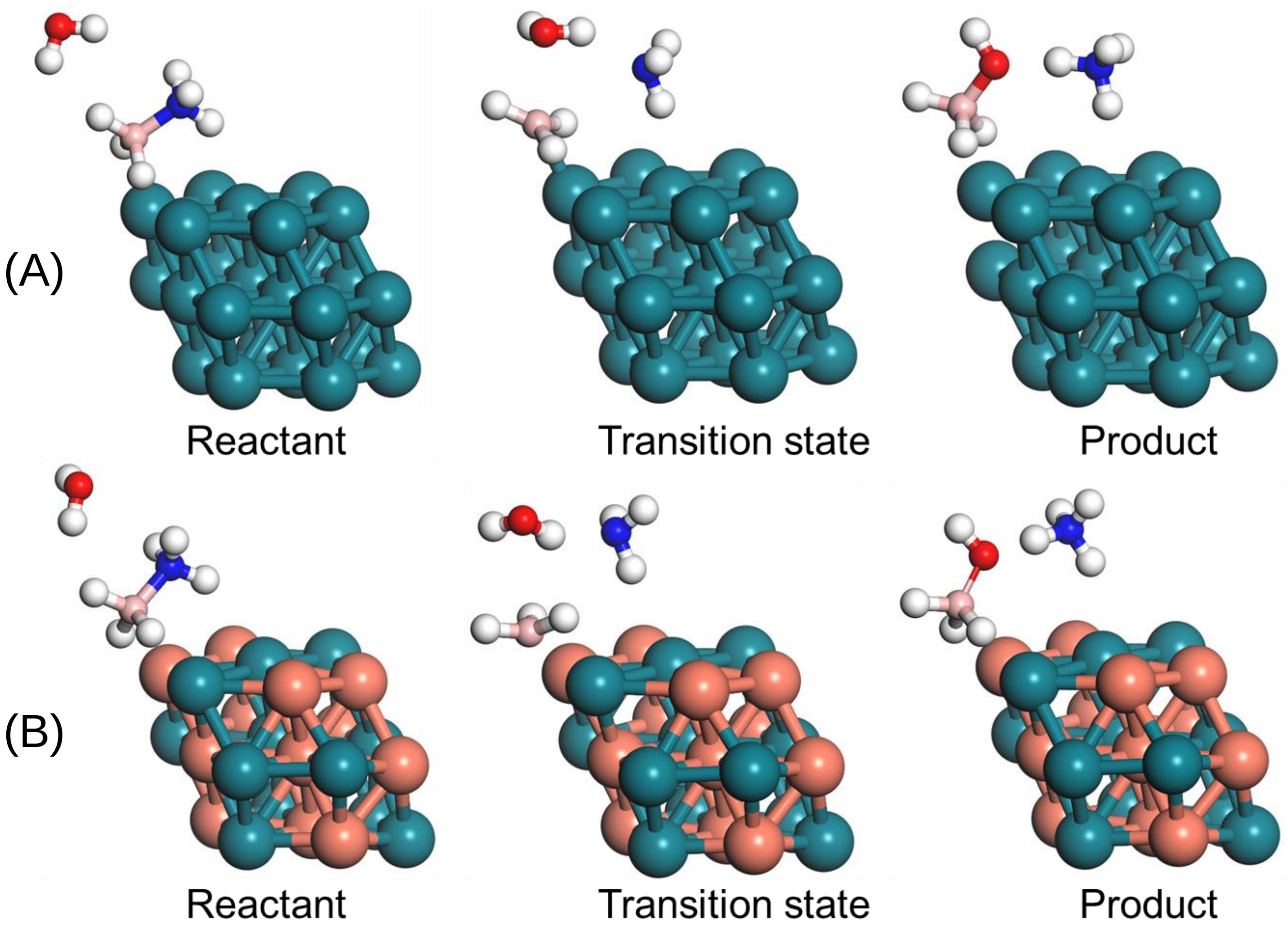

Fig. S7 Calculated model for the rate-limiting step in the AB hydrolysis on the (A) Rh_{24} and (B) $Rh_{12}Cu_{12}$ clusters.

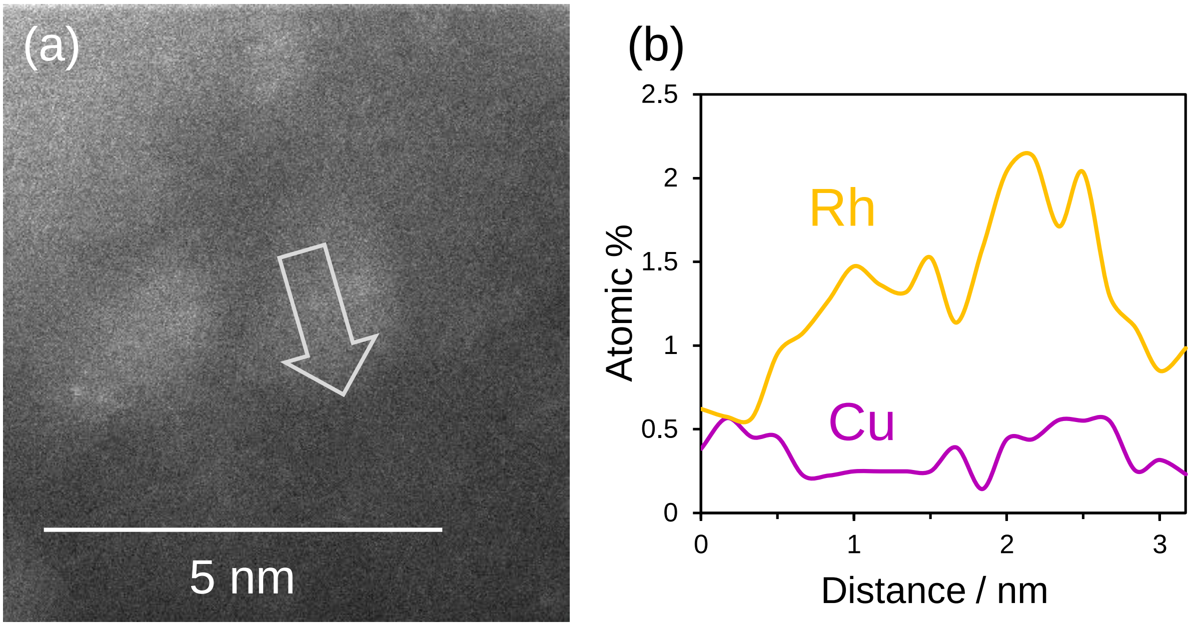

Fig. S8 (a) HAADF-STEM image of small RhCu NPs around 2-3 nm on the RhCu/TiO_2 and (b) EDX line analysis.

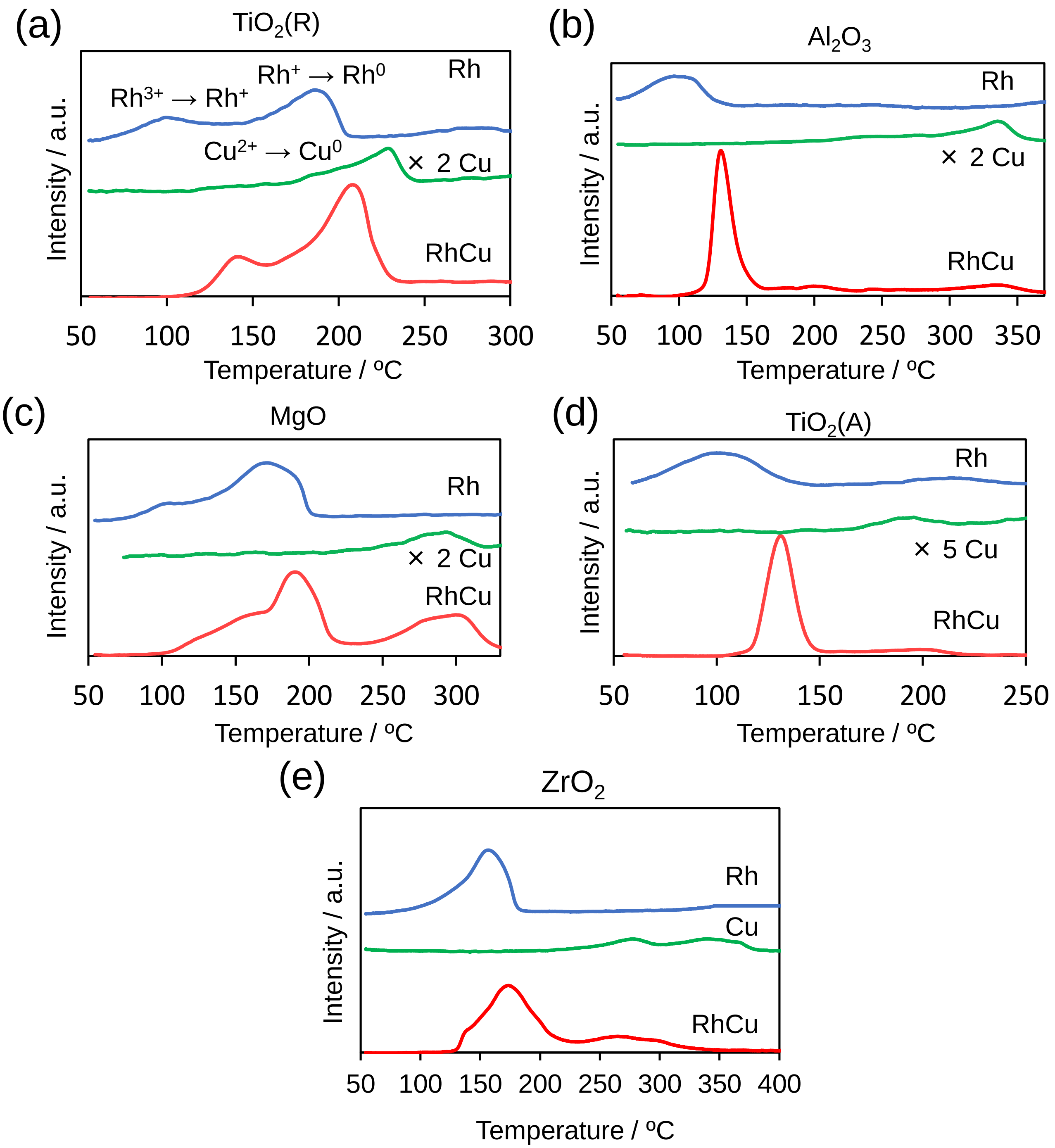

Fig. S9 H_2-TPR profiles for Rh, Cu or RhCu-supported (a) TiO_2(R), (b) Al_2O_3, (c) MgO, (d) TiO_2(A) and (e) ZrO_2 catalysts.

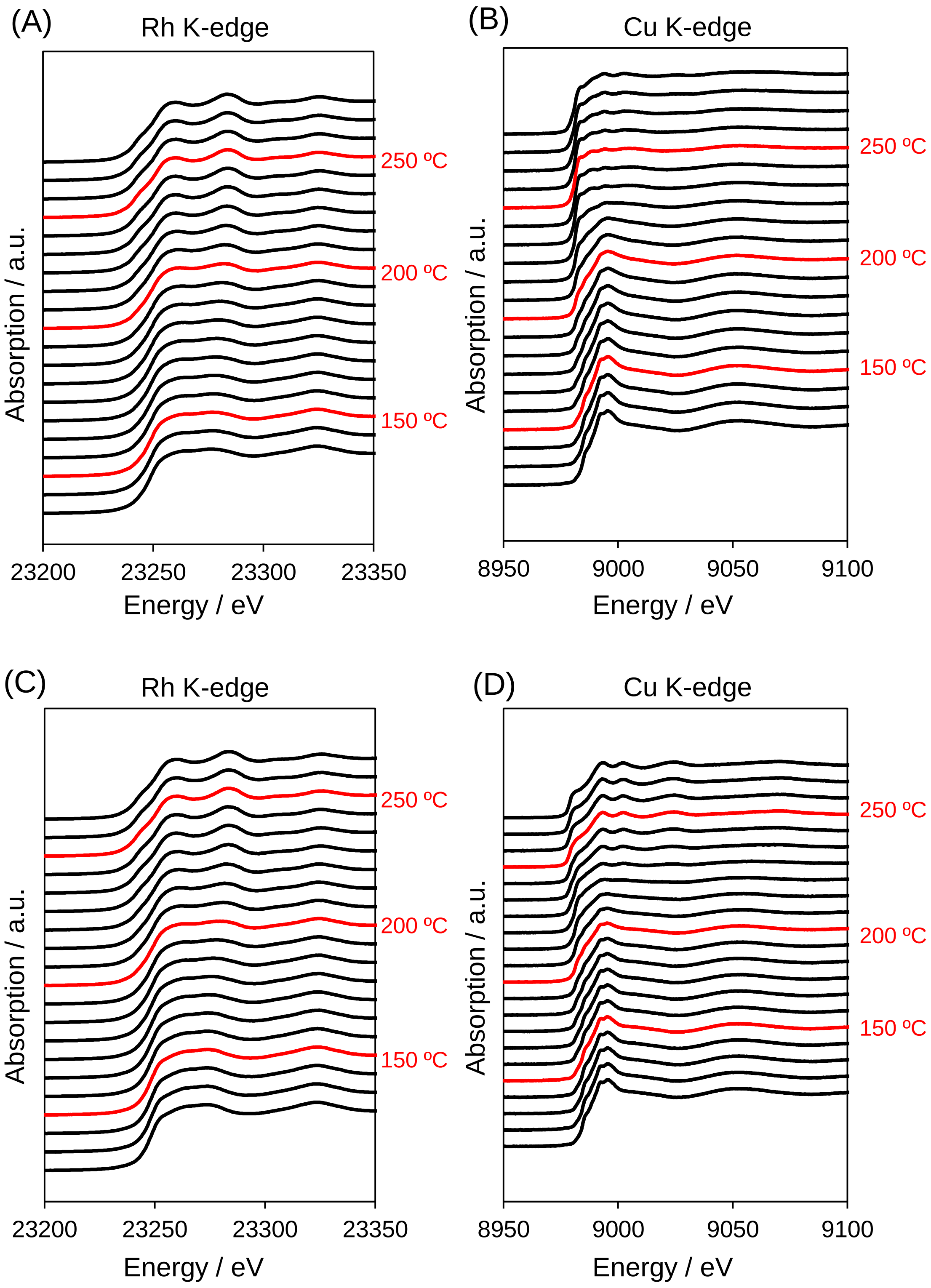

Fig. S10 *In situ* XANES spectra for the (A) Rh K-edge Rh/TiO_2, (B) Cu K-edge Cu/TiO_2, (C) Rh K-edge RhCu/TiO_2 and (D) Cu K-edge RhCu/TiO_2 acquired during reduction under H_2 at elevated temperature.

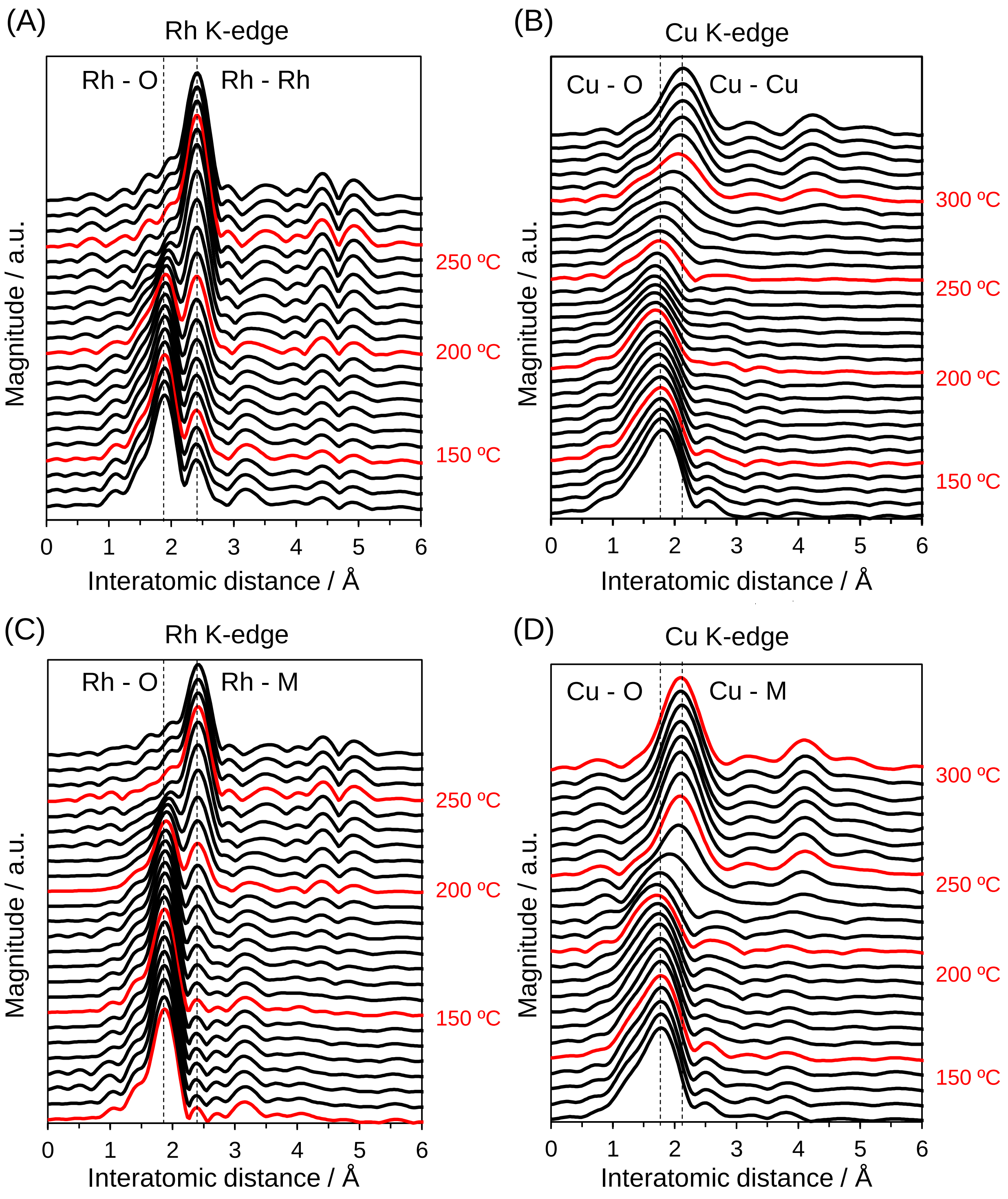

Fig. S11 *In situ* FT-EXAFS spectra for the (A) Rh K-edge Rh/TiO_2, (B) Cu K-edge Cu/TiO_2, (C) Rh K-edge RhCu/TiO_2 and (D) Cu K-edge RhCu/TiO_2 acquired during reduction under H_2 at elevated temperature.

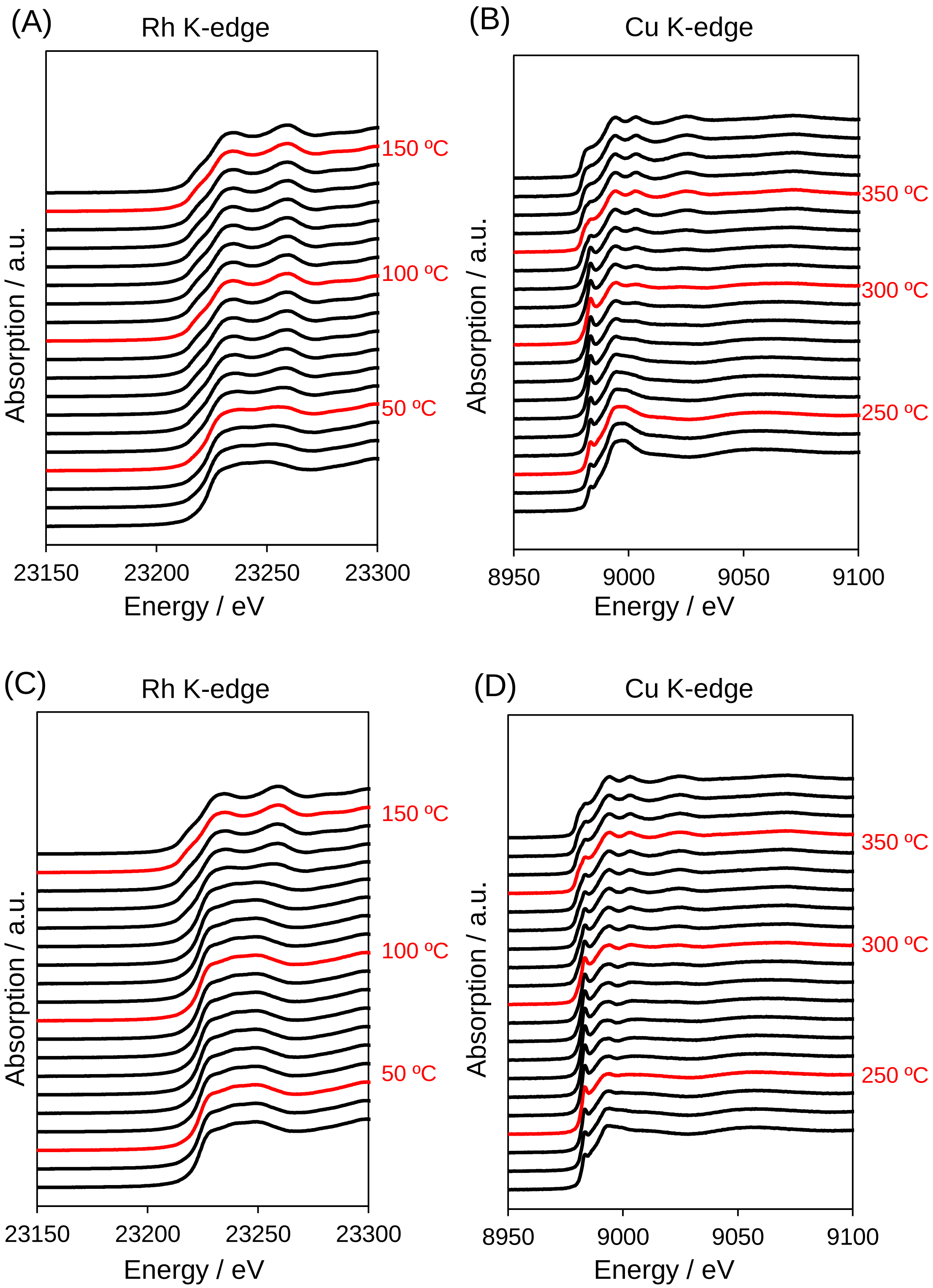

Fig. S12 *In situ* XANES spectra for the (A) Rh K-edge Rh/Al_2O_3, (B) Cu K-edge Cu/Al_2O_3, (C) Rh K-edge RhCu/Al_2O_3 and (D) Cu K-edge RhCu/Al_2O_3 acquired during reduction under H_2 at elevated temperature.

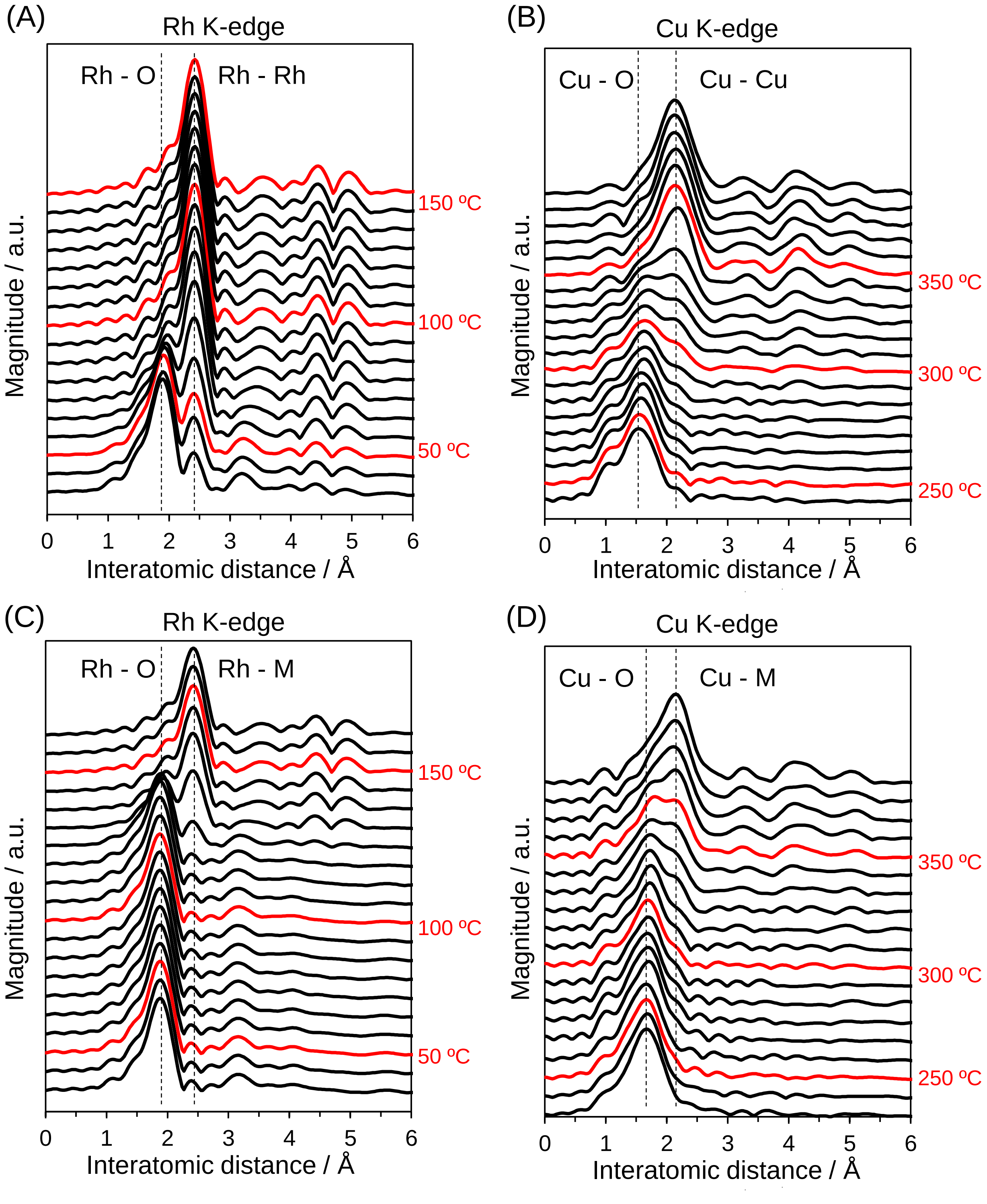

Fig. S13 *In situ* FT-EXAFS spectra for the (A) Rh K-edge Rh/Al_2O_3, (B) Cu K-edge Cu/Al_2O_3, (C) Rh K-edge RhCu/Al_2O_3 and (D) Cu K-edge RhCu/Al_2O_3 acquired during reduction under H_2 at elevated temperature.

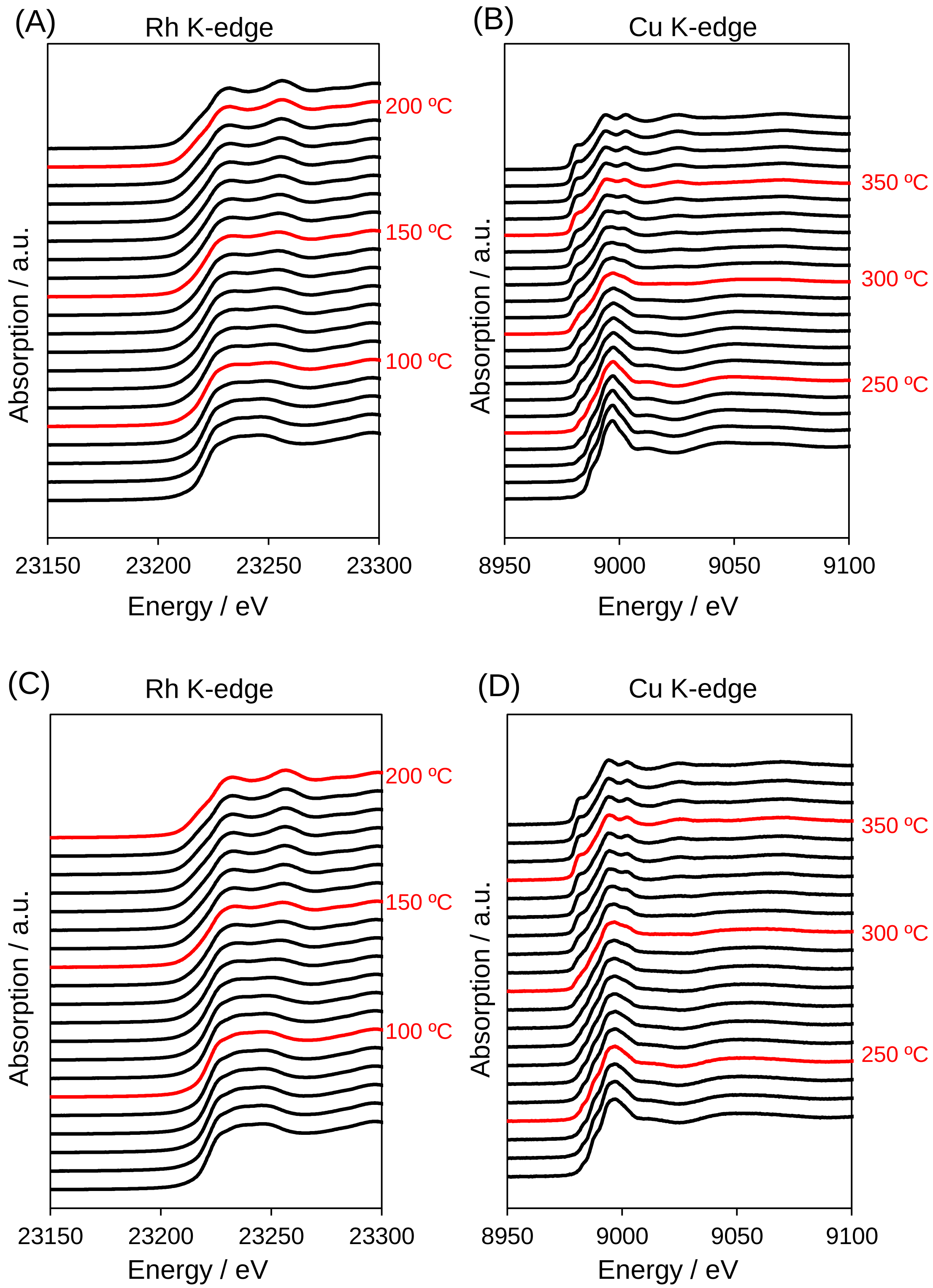

Fig. S14 *In situ* XANES spectra for the (A) Rh K-edge Rh/MgO, (B) Cu K-edge Cu/MgO, (C) Rh K-edge RhCu/MgO and (D) Cu K-edge RhCu/MgO acquired during reduction under H_2 at elevated temperature.

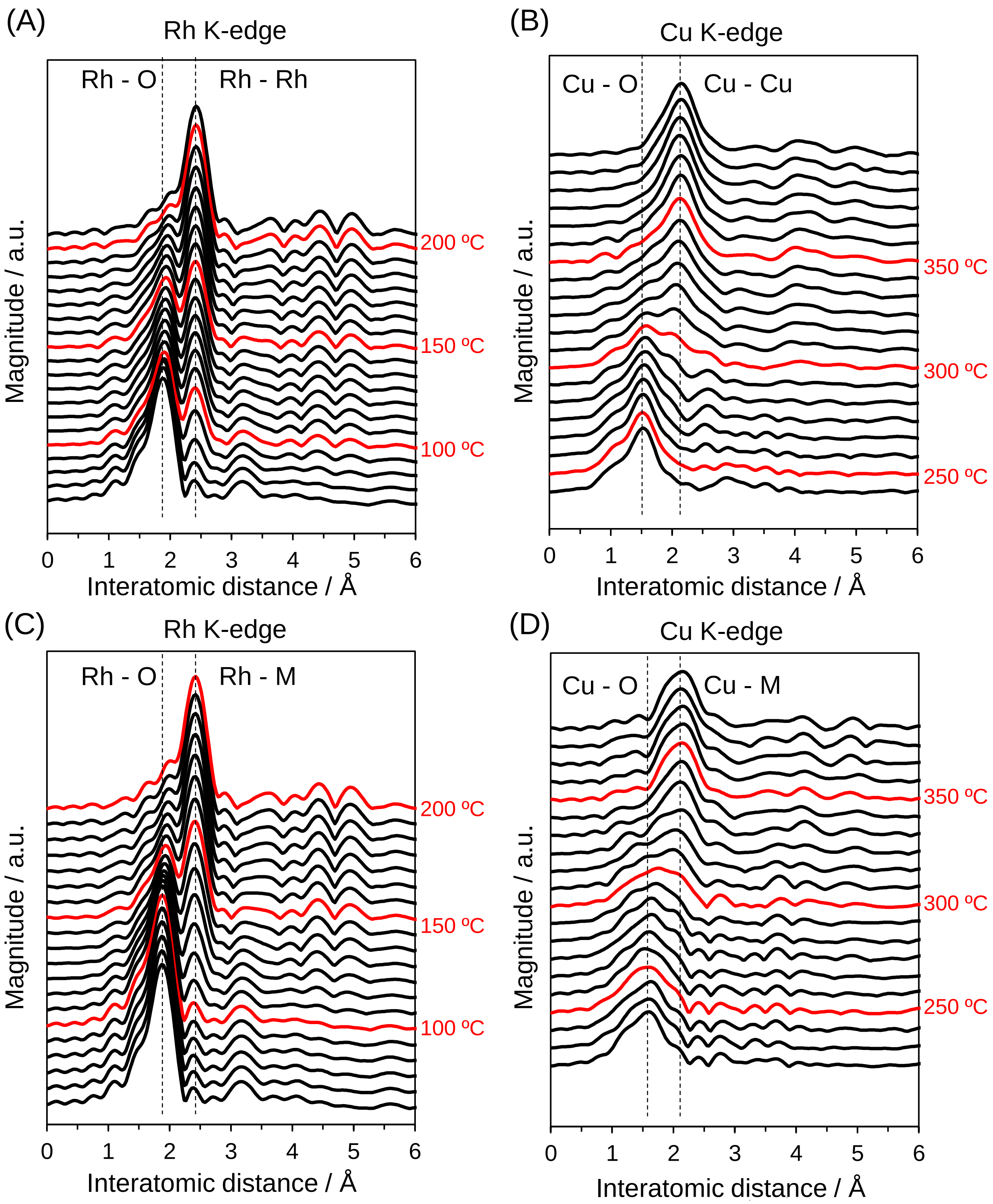

Fig. S15 *In situ* FT-EXAFS spectra for the (A) Rh K-edge Rh/MgO, (B) Cu K-edge Cu/MgO, (C) Rh K-edge RhCu/MgO and (D) Cu K-edge RhCu/MgO acquired during reduction under H_2 at elevated temperature.

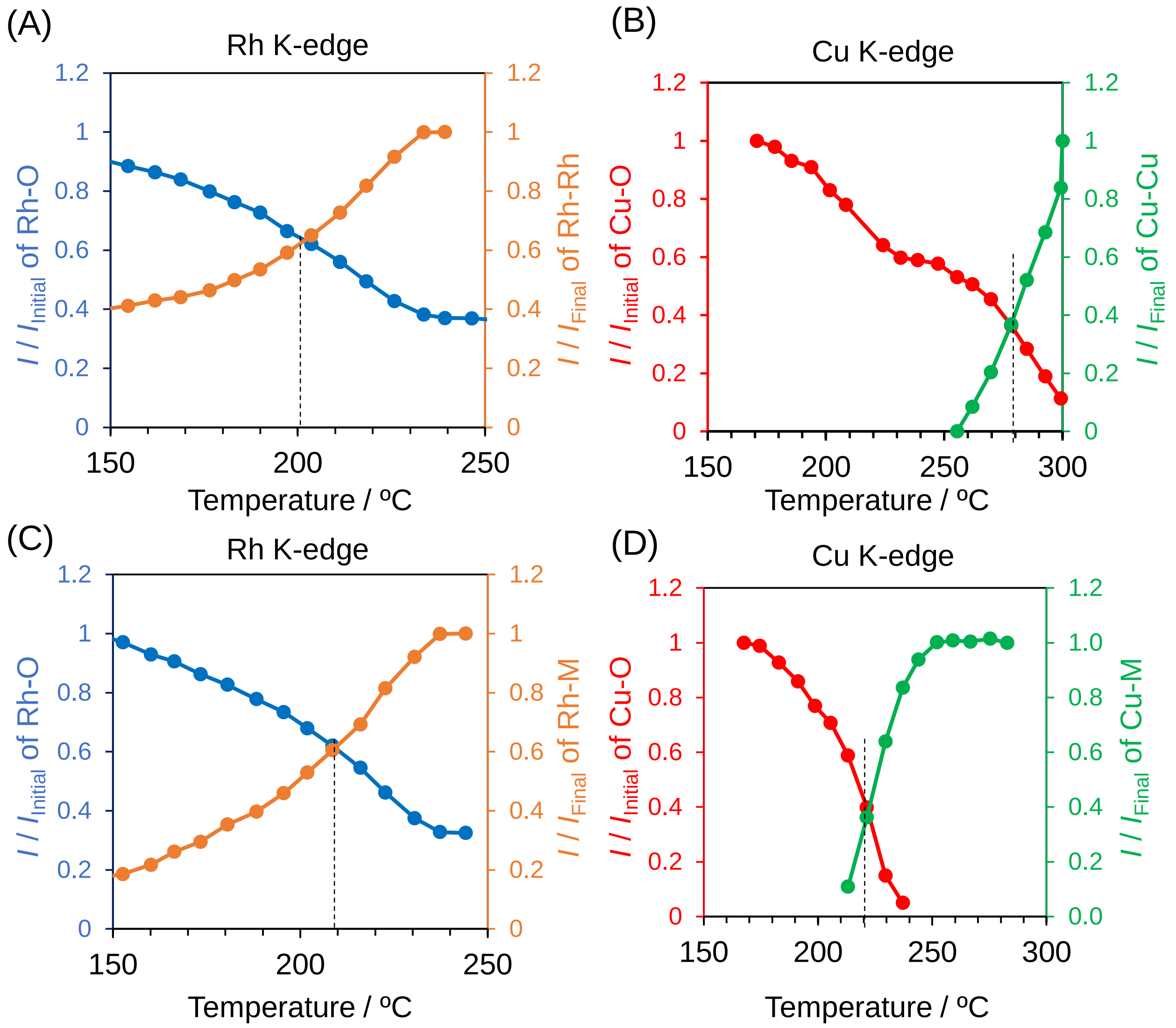

Fig. S16 Variations in the intensities of peaks shown in Fig. S8 attributed to Rh-O, Rh-Rh, Cu-O or Cu-Cu bond during the reduction progress of (A) Rh K-edge Rh/TiO_2, (B) Cu K-edge Cu/TiO_2, (C) Rh K-edge RhCu/TiO_2 and (D) Cu K-edge RhCu/TiO_2. Vertical axis shows relative intensity compared to that of initial data (before H_2 reduction) attributed to Rh-O bond and compared to that of final data (after H_2 reduction) attributed to Rh-Metal(M) bond.

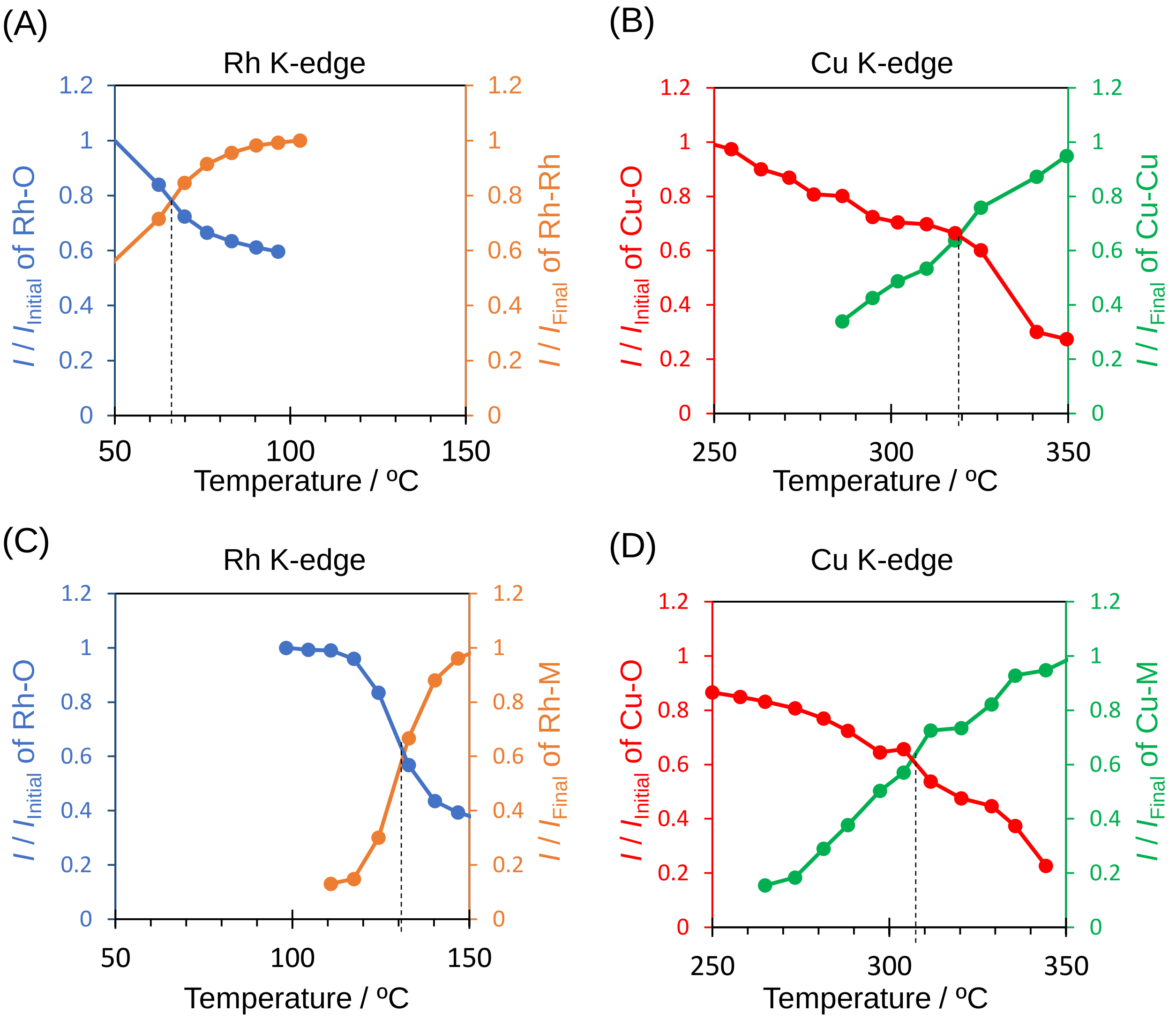

Fig. S17 Variations in the intensities of peaks shown in Fig. S10 attributed to Rh-O, Rh-Rh, Cu-O or Cu-Cu bond during the reduction progress of (A) Rh K-edge Rh/Al_2O_3, (B) Cu K-edge Cu/Al_2O_3, (C) Rh K-edge RhCu/Al_2O_3 and (D) Cu K-edge RhCu/Al_2O_3. Vertical axis shows relative intensity compared to that of initial data (before H_2 reduction) attributed to Rh-O bond and compared to that of final data (after H_2 reduction) attributed to Rh-Metal(M) bond.

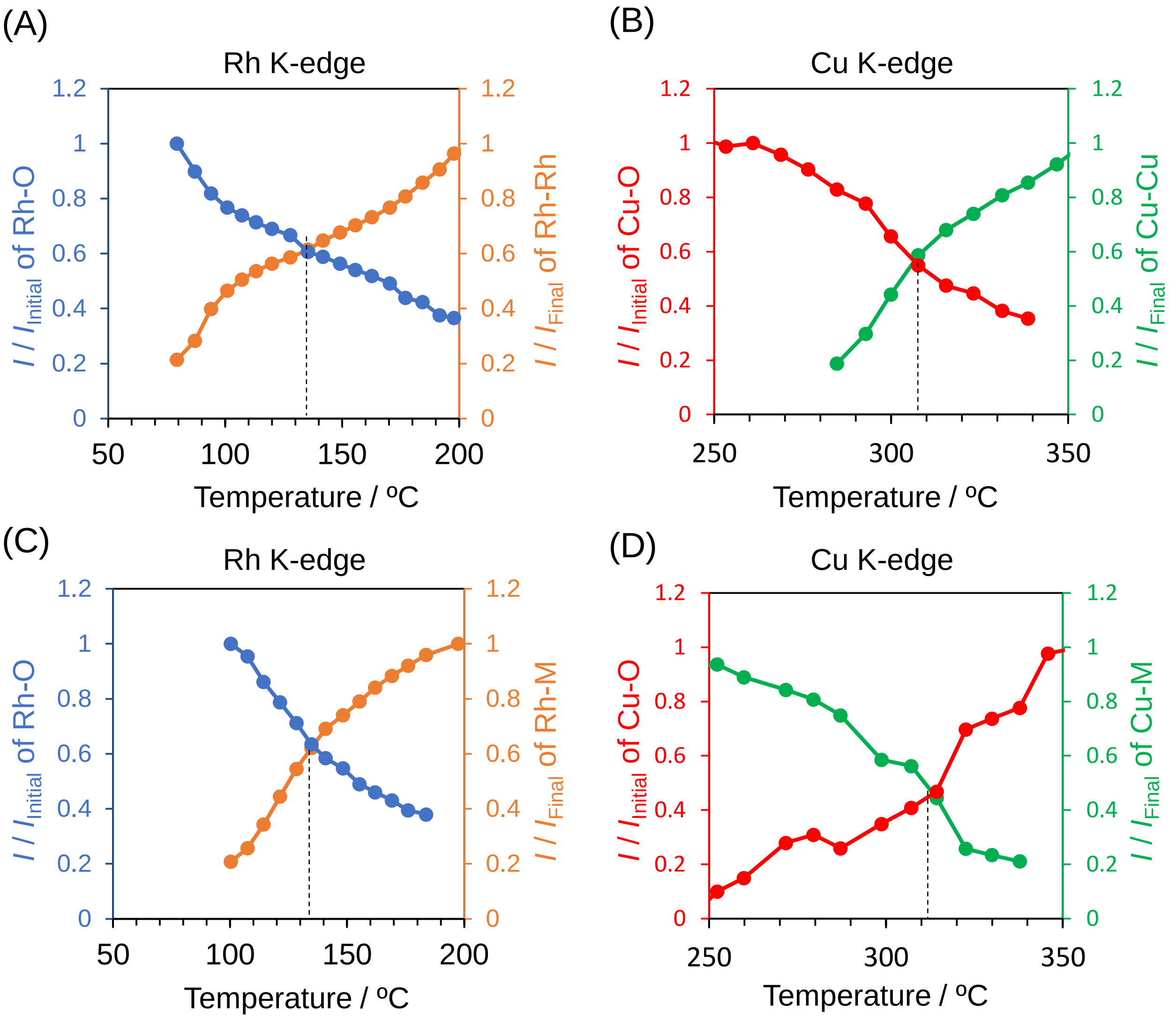

Fig. S18 Variations in the intensities of peaks shown in Fig. S12 attributed to Rh-O, Rh-Rh, Cu-O or Cu-Cu bond during the reduction progress of (A) Rh K-edge Rh/MgO, (B) Cu K-edge Cu/MgO, (C) Rh K-edge RhCu/MgO and (D) Cu K-edge RhCu/MgO. Vertical axis shows relative intensity compared to that of initial data (before H_2 reduction) attributed to Rh-O bond and compared to that of final data (after H_2 reduction) attributed to Rh-Metal(M) bond.

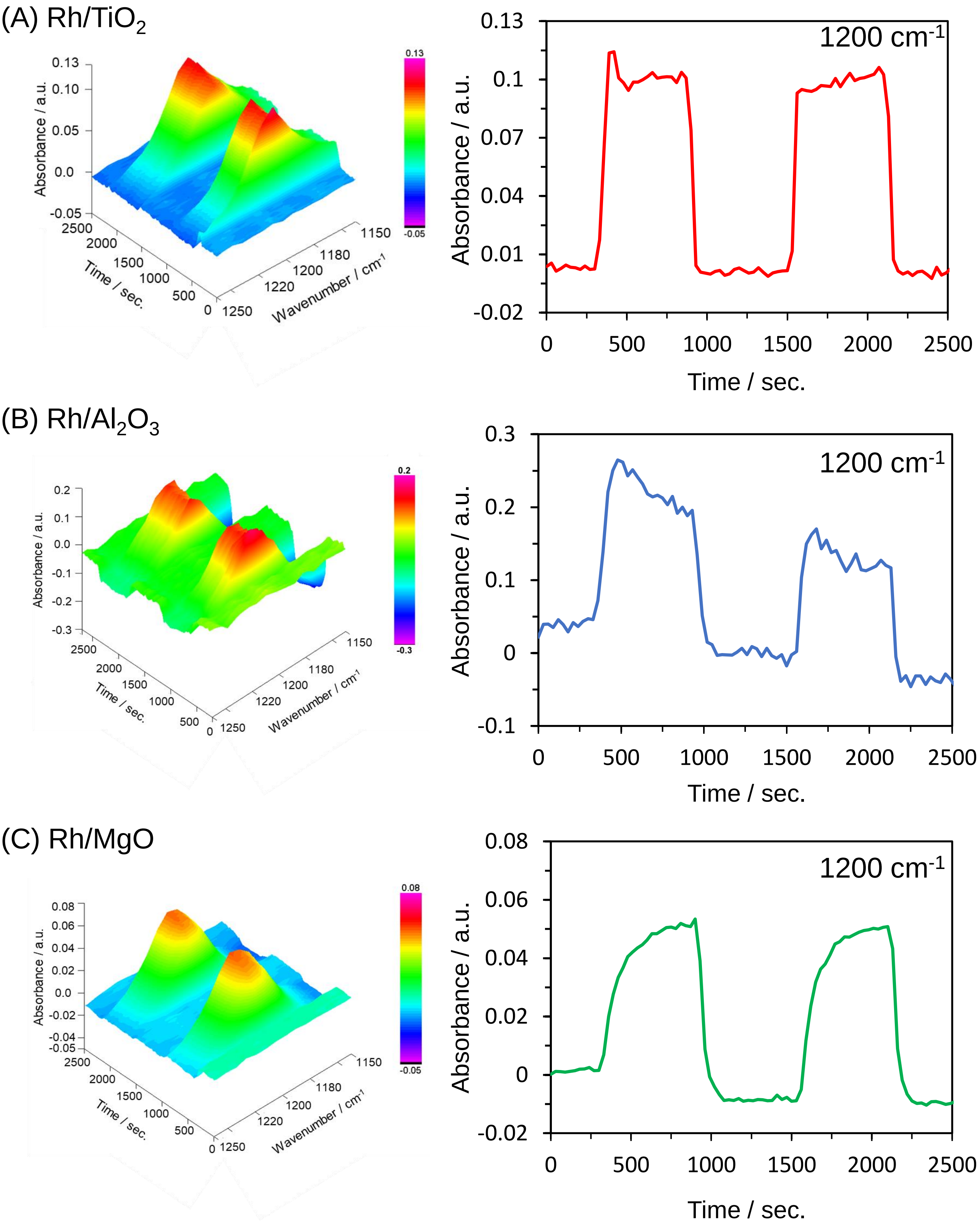

Fig. S19 time courses of the changes in the intensity of the peak at 1200 cm^{-1} attributed to the δ_{D-O-D} stretching vibration as obtained from *in situ* FTIR spectra acquired during the H_2 and D2 gas exchange sequence over Rh supported (a) TiO_2, (b) Al_2O_3 and (c) MgO.

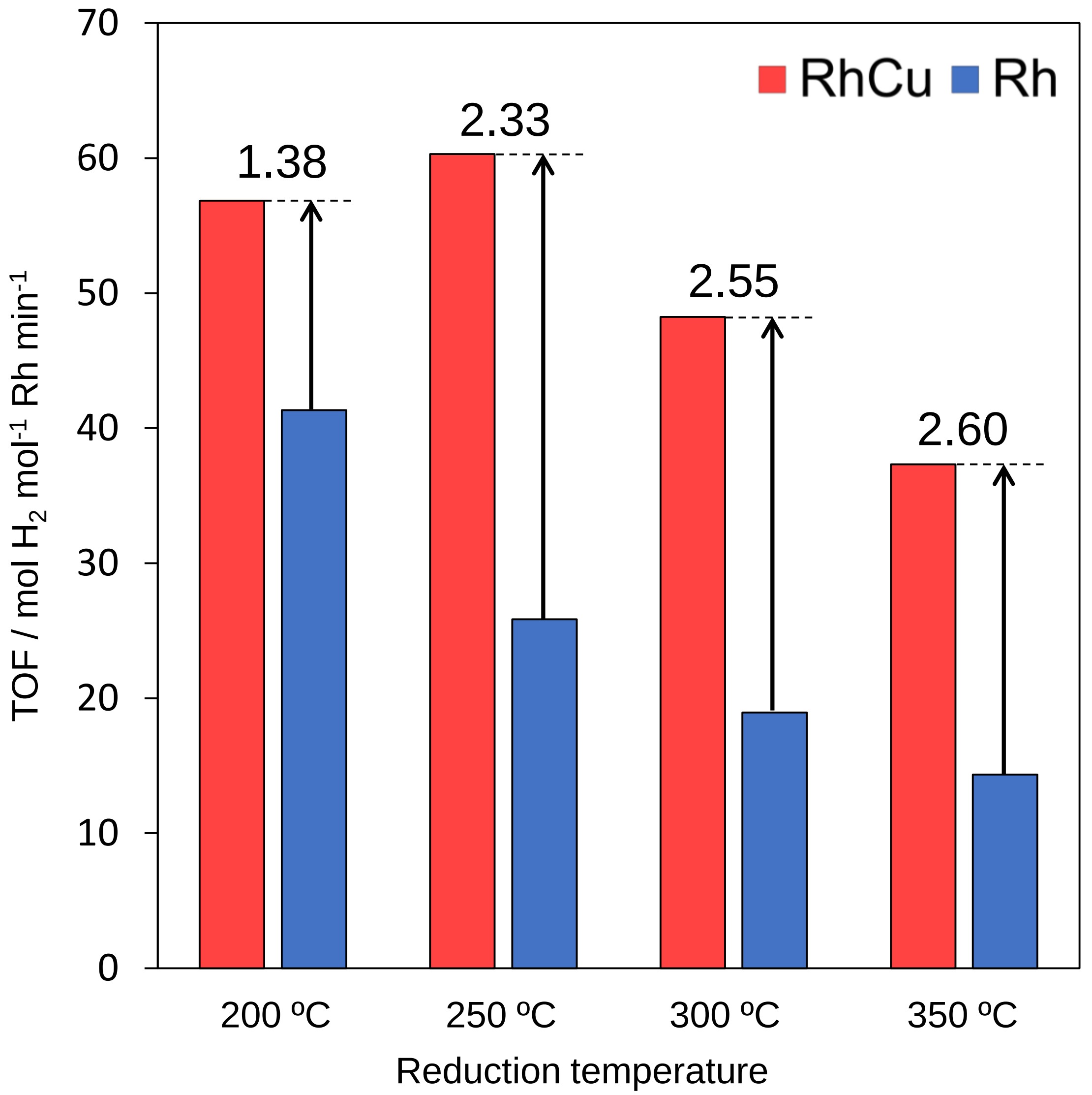

Fig. S20 Difference of activity improvement of RhCu/TiO_2 compared to Rh/TiO_2 depending on the reduction temperature of each catalyst (catalytic conditions: catalyst 20 mg, 10 mL 0.2 M aqueous AB solution, 30 ºC).

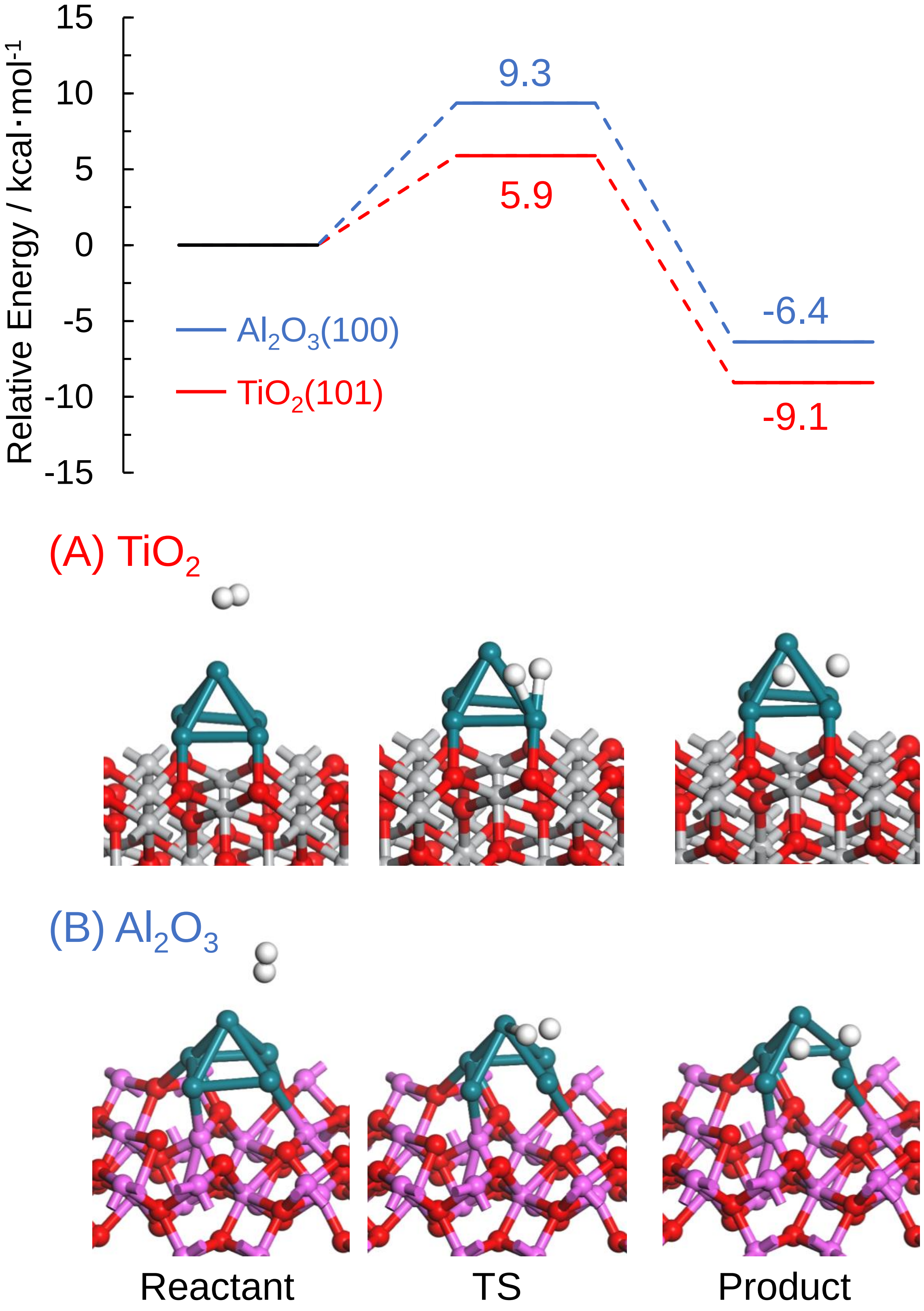

Fig. S21 Energy profiles and calculated model for the H_2 cleavage on Rh_5 cluster (Step 1) on the TiO_2 and Al_2O_3.

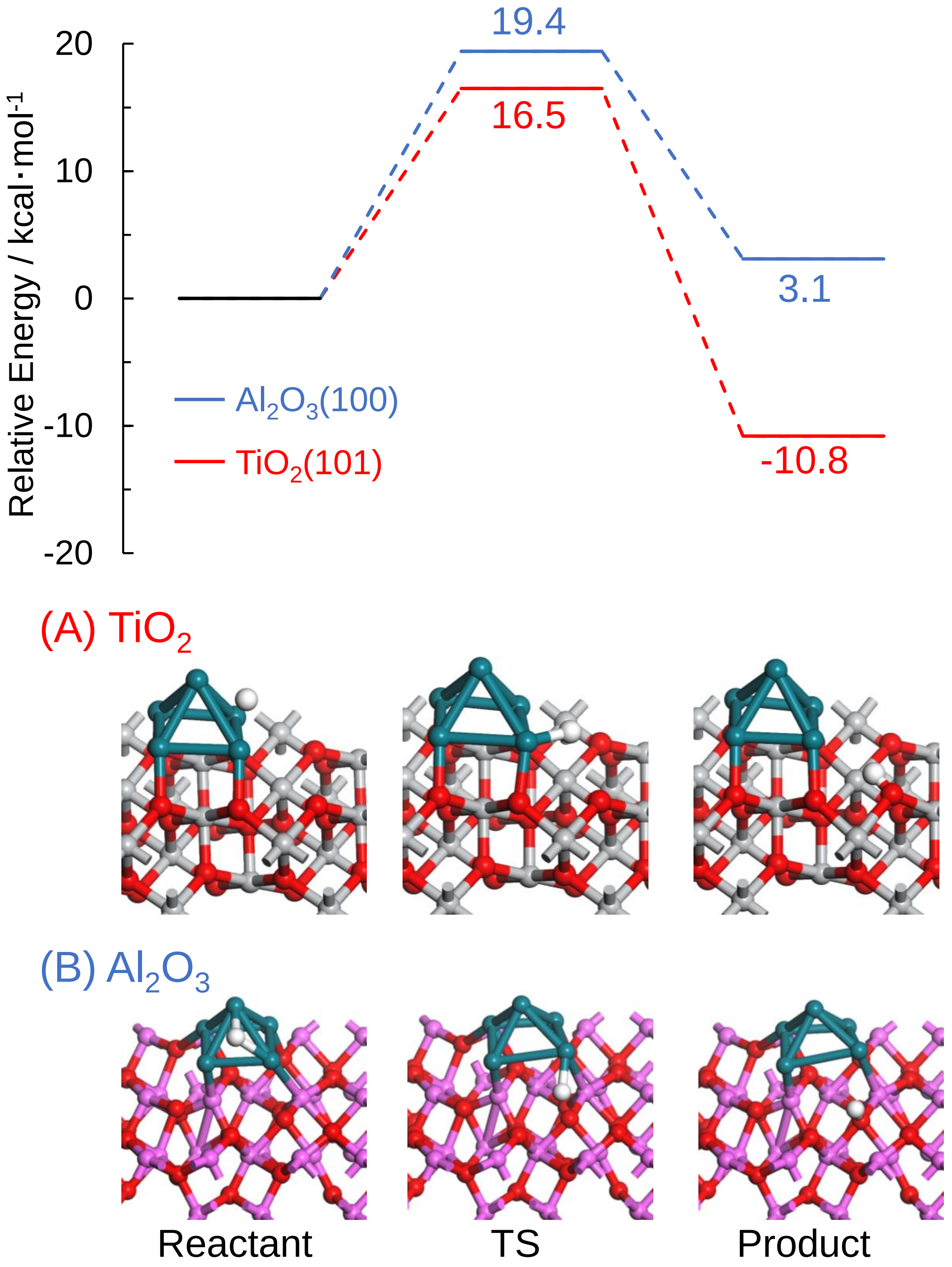

Fig. S22 Energy profiles and calculated model for the H atom transfer from Rh_5 cluster to each support (Step 2) on the TiO_2 and Al_2O_3.

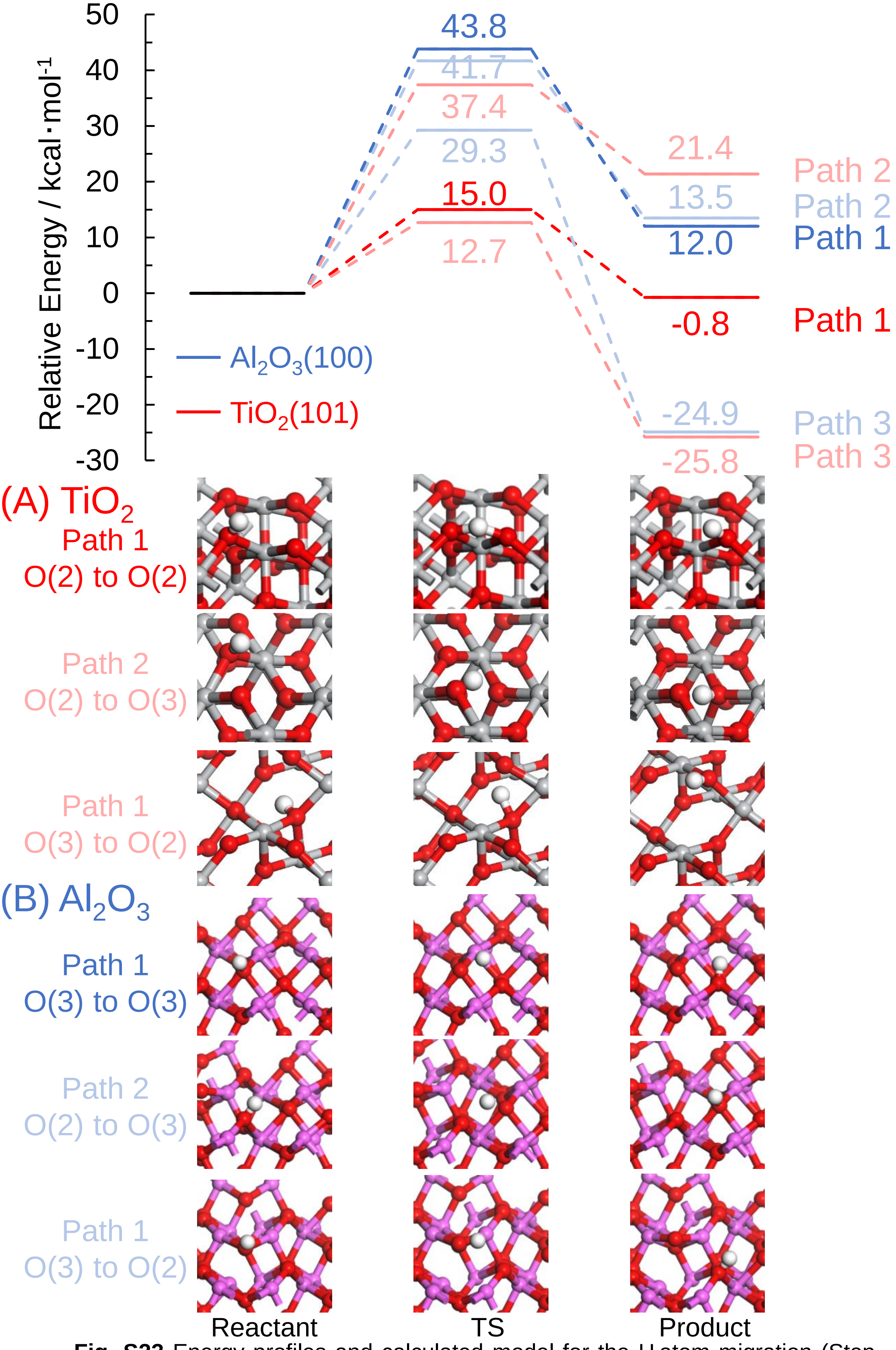

Fig. S23 Energy profiles and calculated model for the H atom migration (Step 3) on the TiO_2 and Al_2O_3.

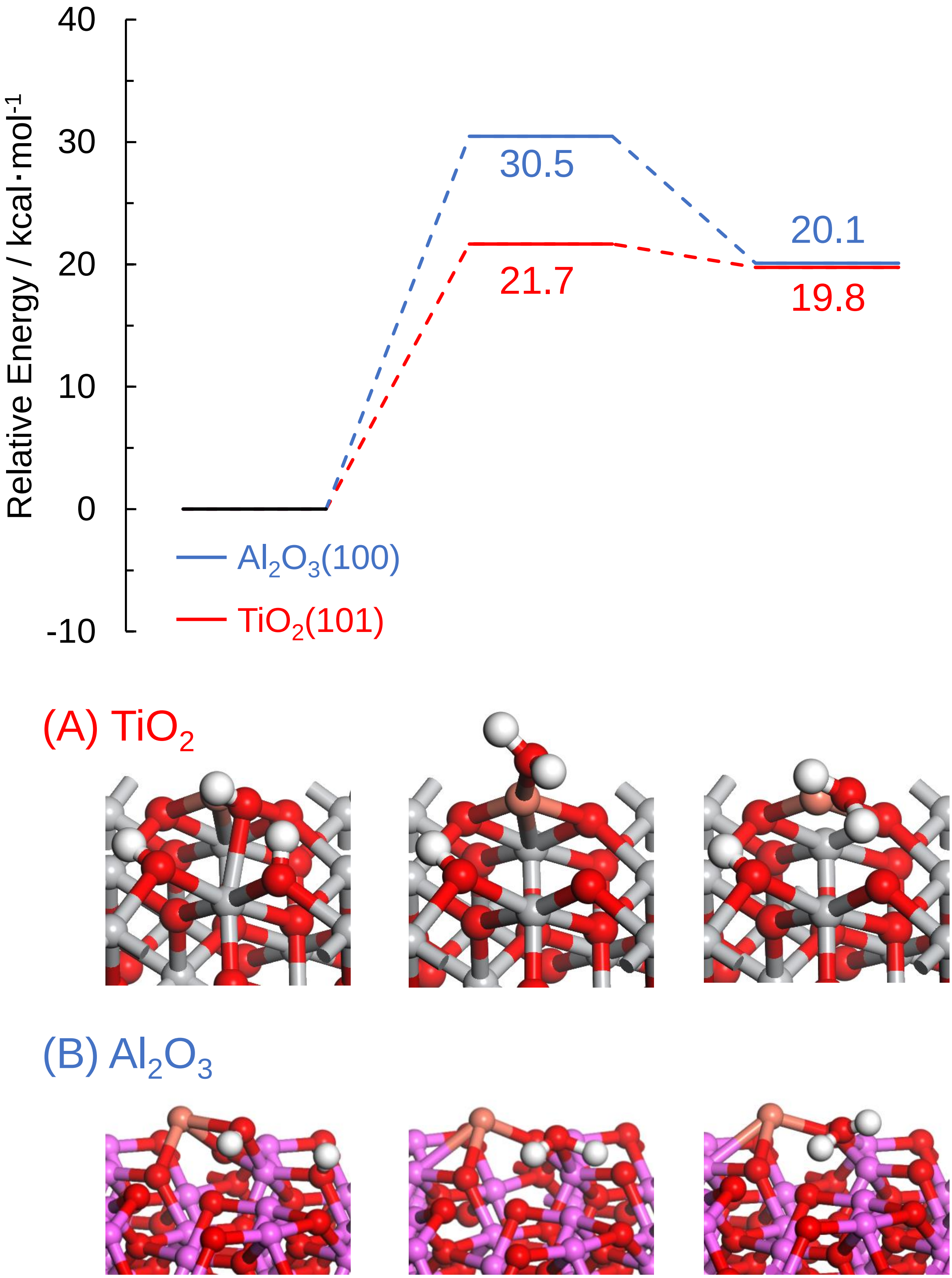

Fig. S24 Energy profiles and calculated model for the reduction of Cu species by spilled H atom (Step 4) on the TiO_2 and Al_2O_3.

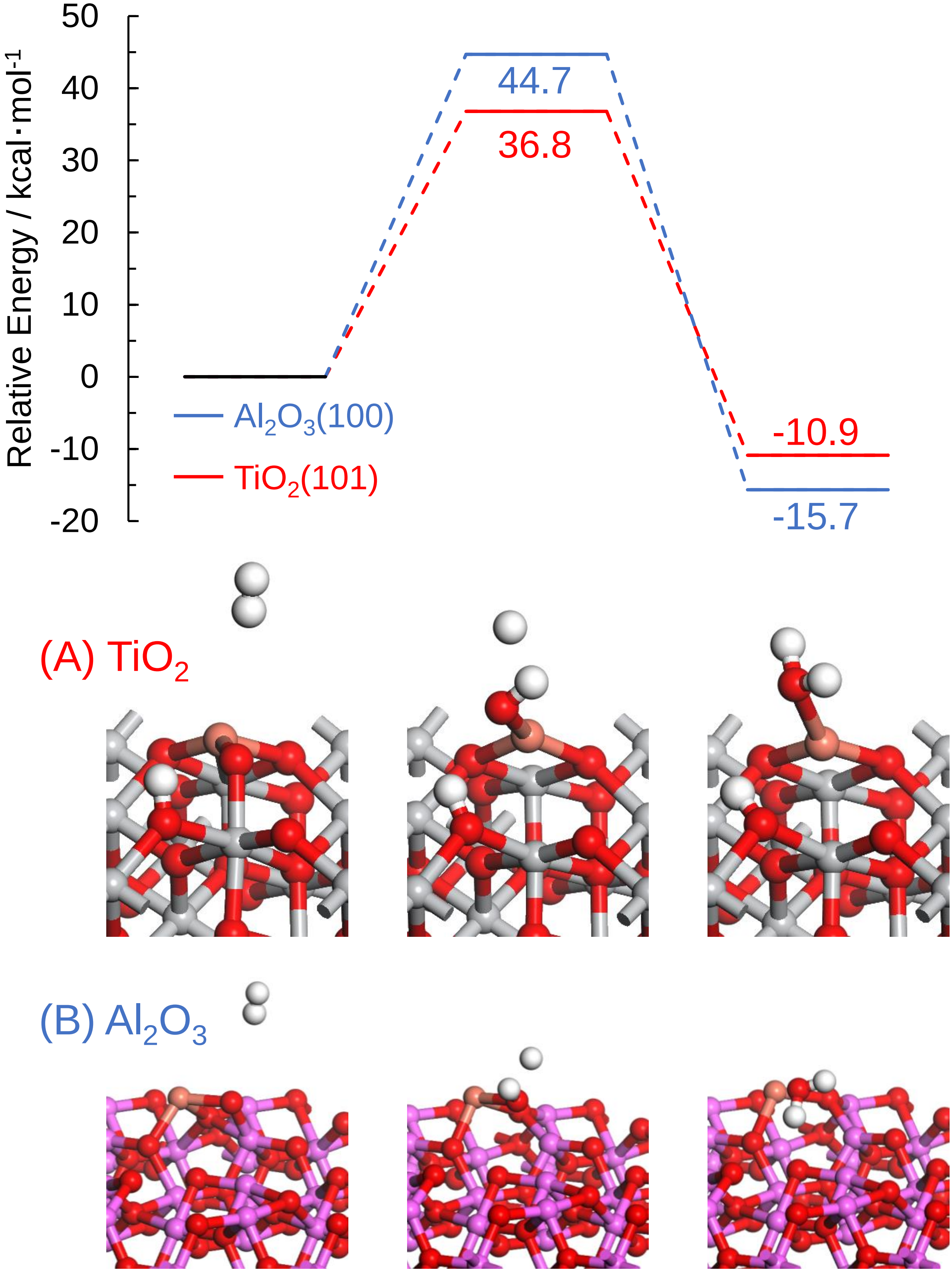

Fig. S25 Energy profiles and calculated model for the reduction of Cu species by vapor H_2 on the TiO_2 and Al_2O_3.

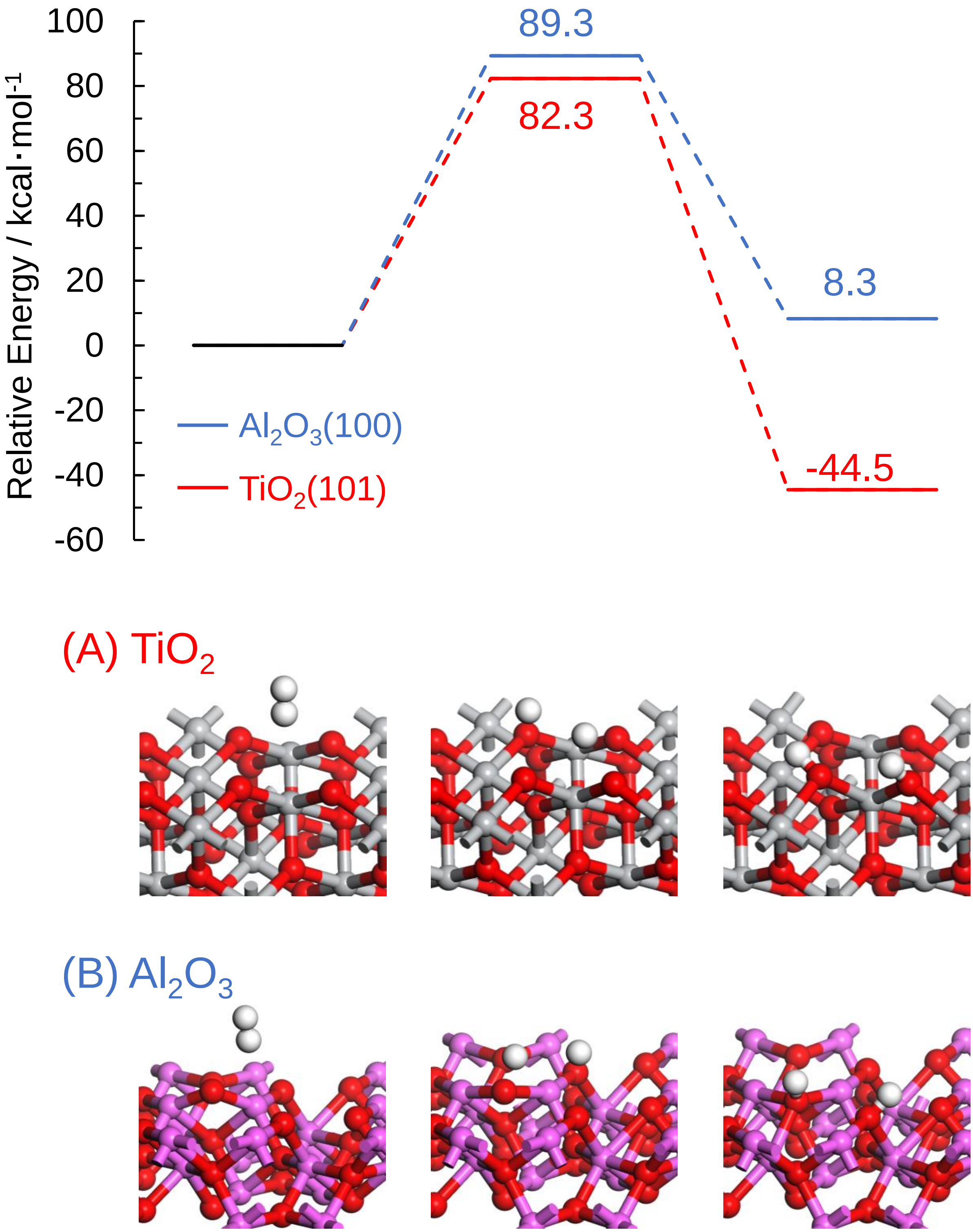

Fig. S26 Energy profiles and calculated model for the H_2 cleavage on each support without Rh_5 cluster on the TiO_2 and Al_2O_3.

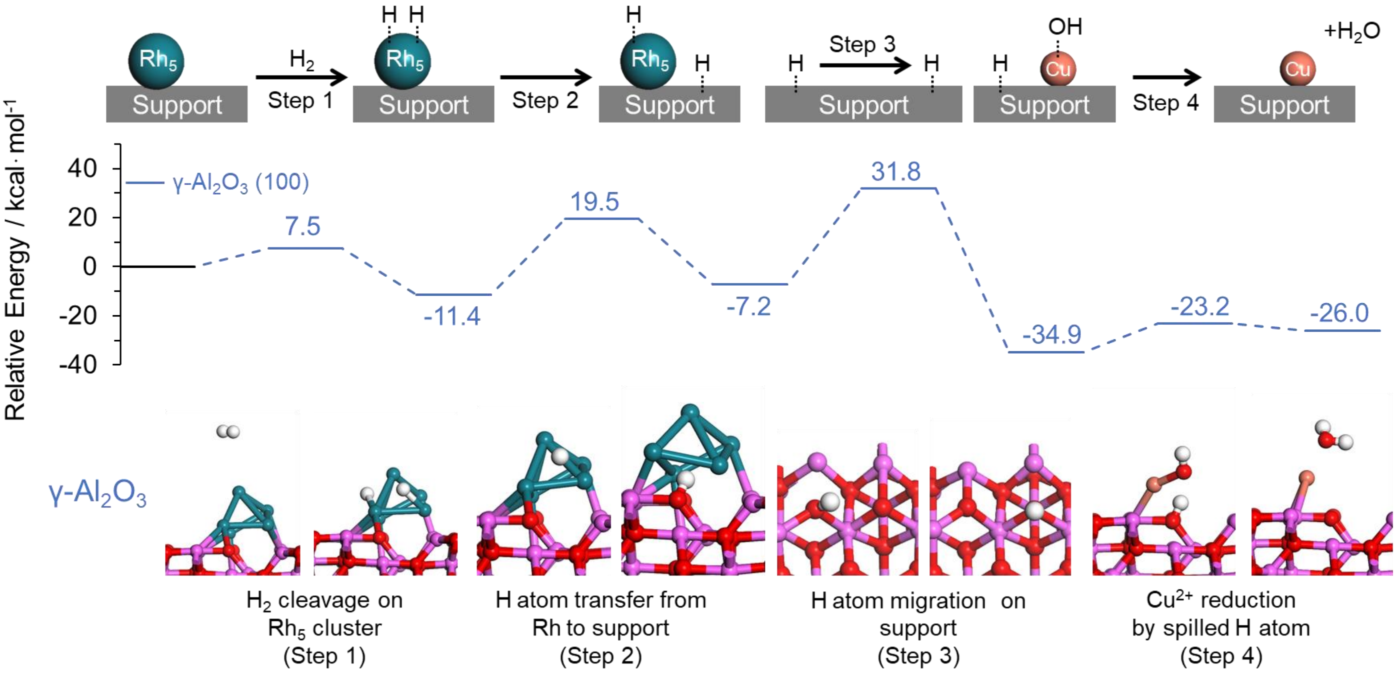

Figure S27. Theoretical pathway for the reduction of Cu species on the γ-Al_2O_3 supports assisted by hydrogen spillover.

Table S1. Activation energies for various steps during the reduction of Cu^{2+} species assisted by Hydrogen spillover on γ-Al_2O_3 (100).

	Activation Energy / kcal·mol^{-1}			
	H_2 cleavage on Rh_5 cluster	H atom transfer from Rh_5 cluster to support	H atom migration on support	Cu^{2+} reduction by spilled H atom
γ-Al_2O_3 (100)	7.5	30.9	38.9	11.7
